# Supplementary material for: 2,5-Diketopiperazine Derivatives as Potential Anti-Influenza (H5N2) Agents: Synthesis, Biological Evaluation, and Molecular Docking Study
Source: Molecules. 2022 Jun 29;27(13):4200. doi: 10.3390/molecules27134200 (PMC9268516; doi:10.3390/molecules27134200)
Supplement: Supplementary file 1 [file molecules-27-04200-s001.zip › molecules-1726820-supplementary.pdf]

Supplementary Materials

2,5-Diketopiperazine Derivatives as Potential Anti-Influenza (H5N2) Agents: Synthesis, Biological Evaluation, and Molecular Docking Study

Chanakan Winyakul [1], Weerachai Phutdhawong [2], Poomipat Tamdee [1], Jitnapa Sirirak [1], Thongchai Taechowisan [3] and Waya S. Phutdhawong [1,*]

[1] Department of Chemistry, Faculty of Science, Silpakorn University, Nakorn Pathom 73000, Thailand; chanakanwnkn@gmail.com (C.W.), tamdee_p@silpakorn.edu (P.T.), jitnapasirirak@gmail.com (J.S.)
[2] Department of Chemistry, Faculty of Liberal Arts and Science, Kasetsart University, Kamphaengsaen Campus, Nakorn Pathom 73140, Thailand; phutdhawong@gmail.com
[3] Department of Microbiology, Faculty of Science, Silpakorn University, Nakorn Pathom 73000, Thailand; tewson84@hotmail.com
* Correspondence: phutdhawong_w@su.ac.th; Tel.: +66-34-255797

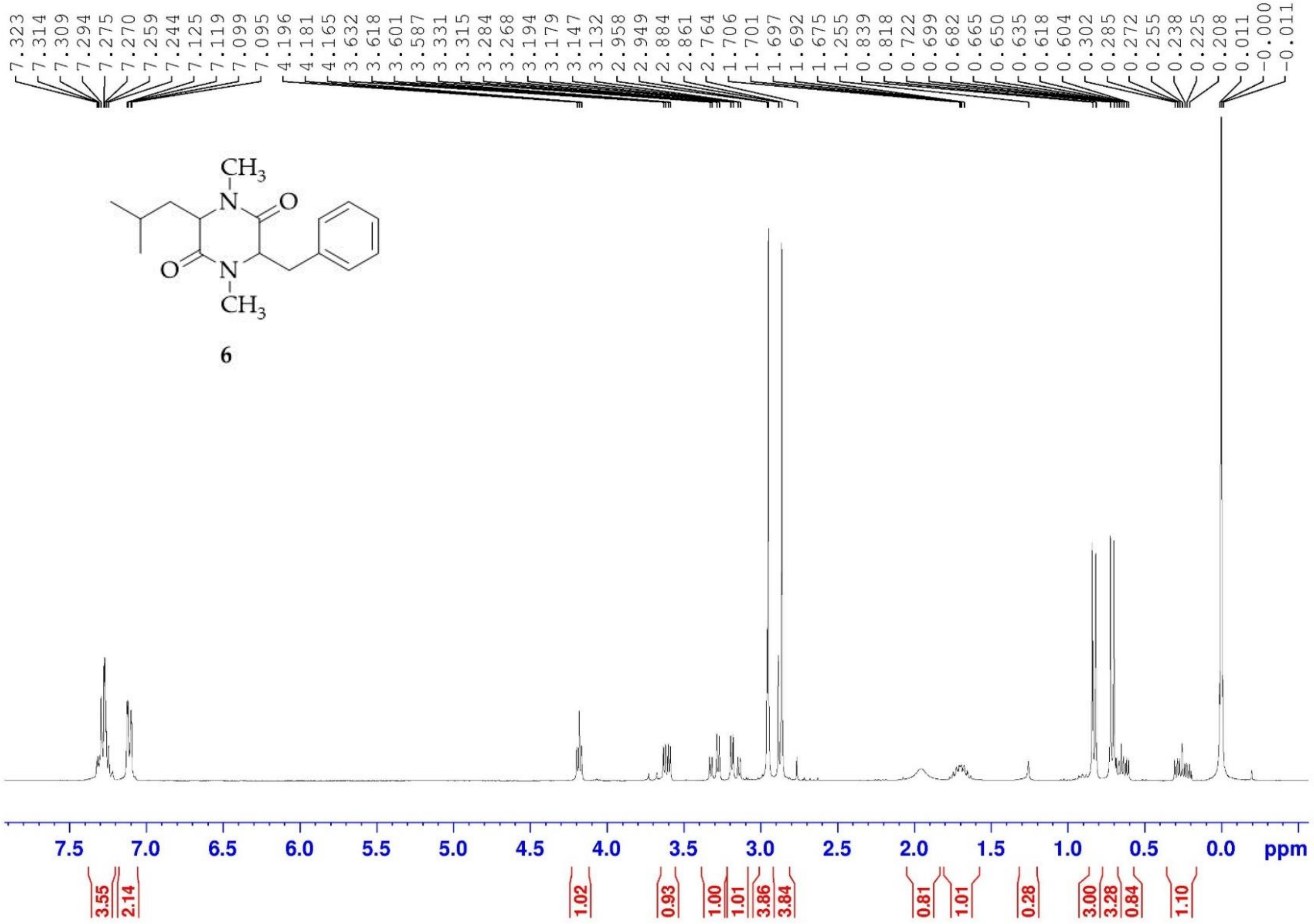

Figure S1. ^{1}H-NMR of the (*3S*,*6S*)-3-Benzyl-6-isobutyl-1,4-dimethyl-2,5-diketopiperazine (**6**).

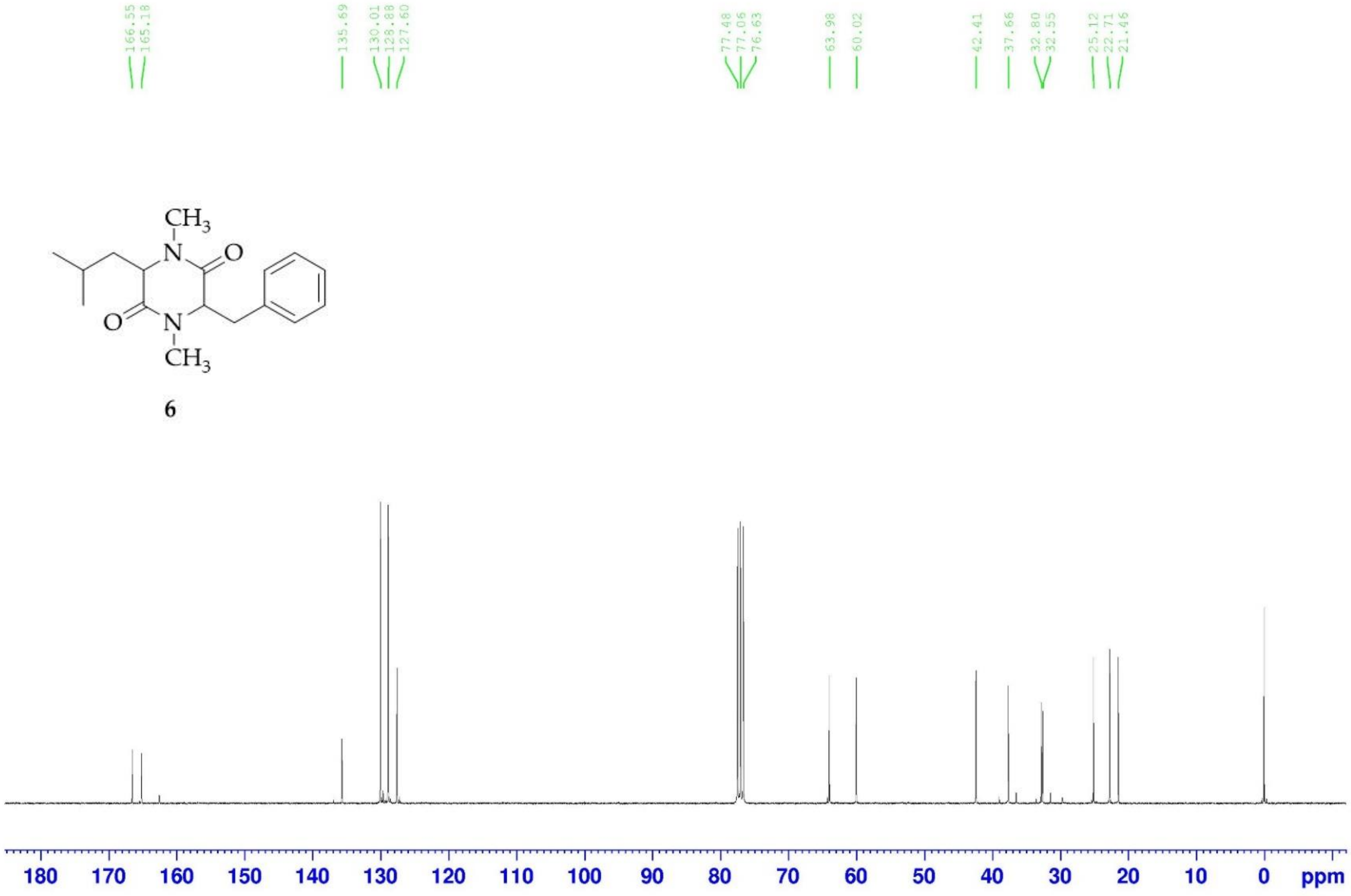

Figure S2. ^{13}C-NMR of the (*3S*,*6S*)-3-Benzyl-6-isobutyl-1,4-dimethyl-2,5-diketopiperazine (**6**).

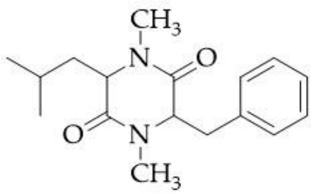

6

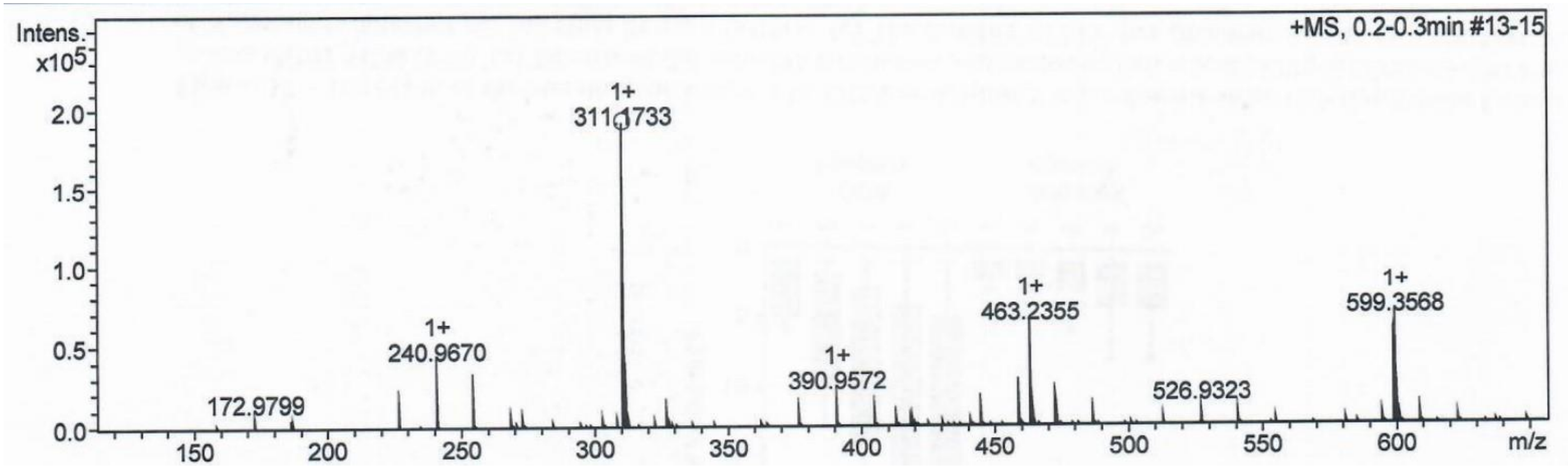

Figure S3. HRMS of the (*3S*,*6S*)-3-Benzyl-6-isobutyl-1,4-dimethyl-2,5-diketopiperazine (**6**).

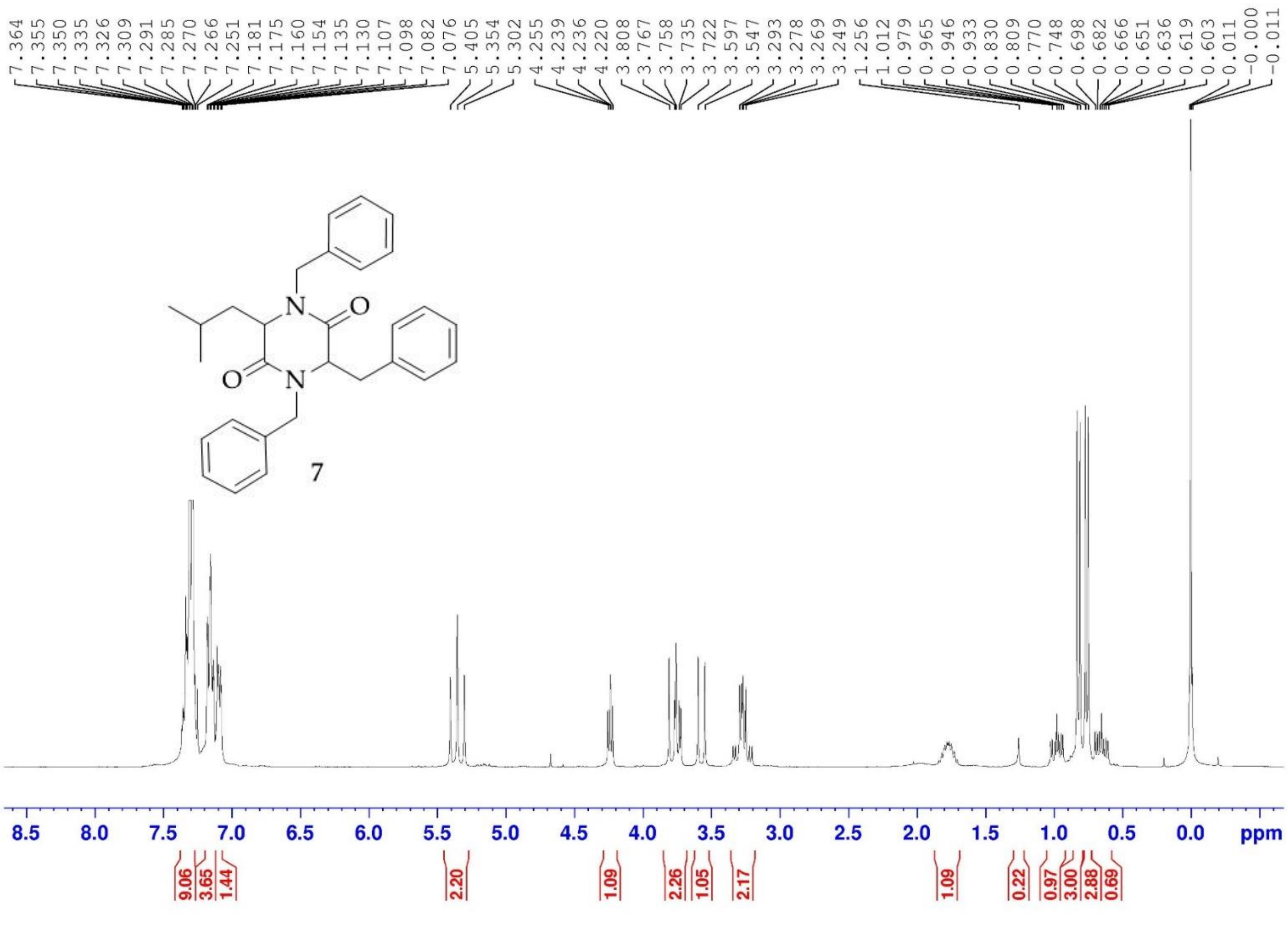

Figure S4. ^{1}H-NMR of the (*3S*,*6S*)-1,3,4-Tribenzyl-6-isobutyl-2,5-diketopiperazine (**7**).

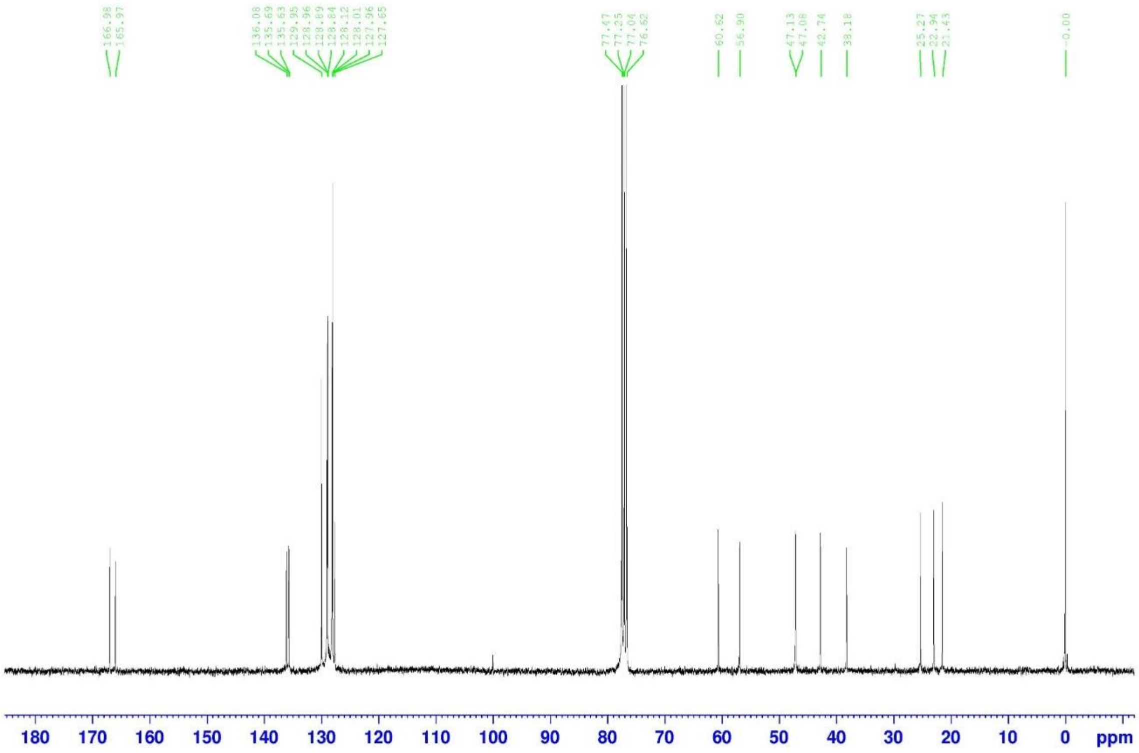

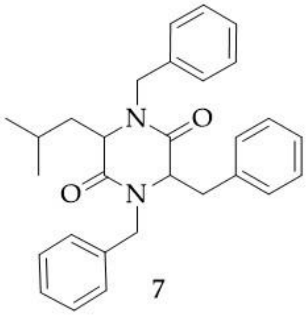

7

Figure S5. ^{13}C-NMR of the (*3S*,*6S*)-1,3,4-Tribenzyl-6-isobutyl-2,5-diketopiperazine (**7**).

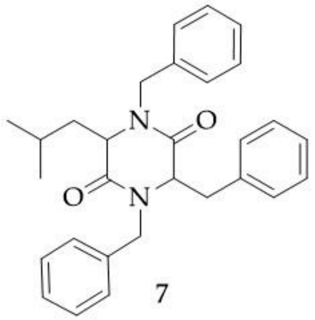

7

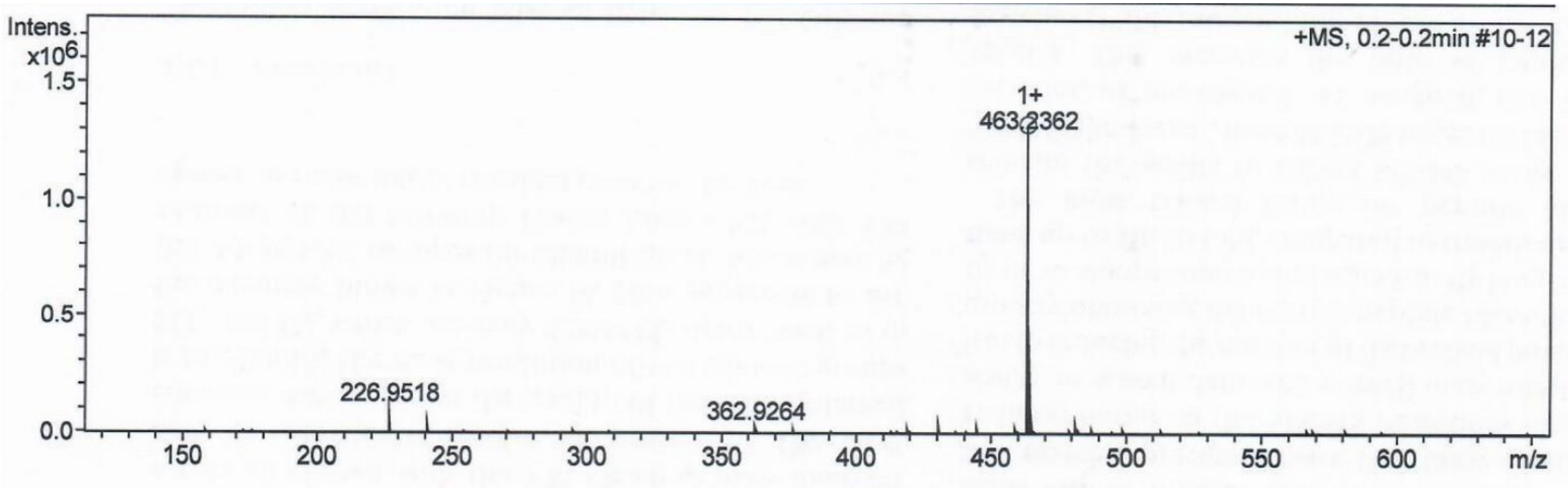

Figure S6. HRMS of the (*3S*,*6S*)-1,3,4-Tribenzyl-6-isobutyl-2,5-diketopiperazine (**7**).

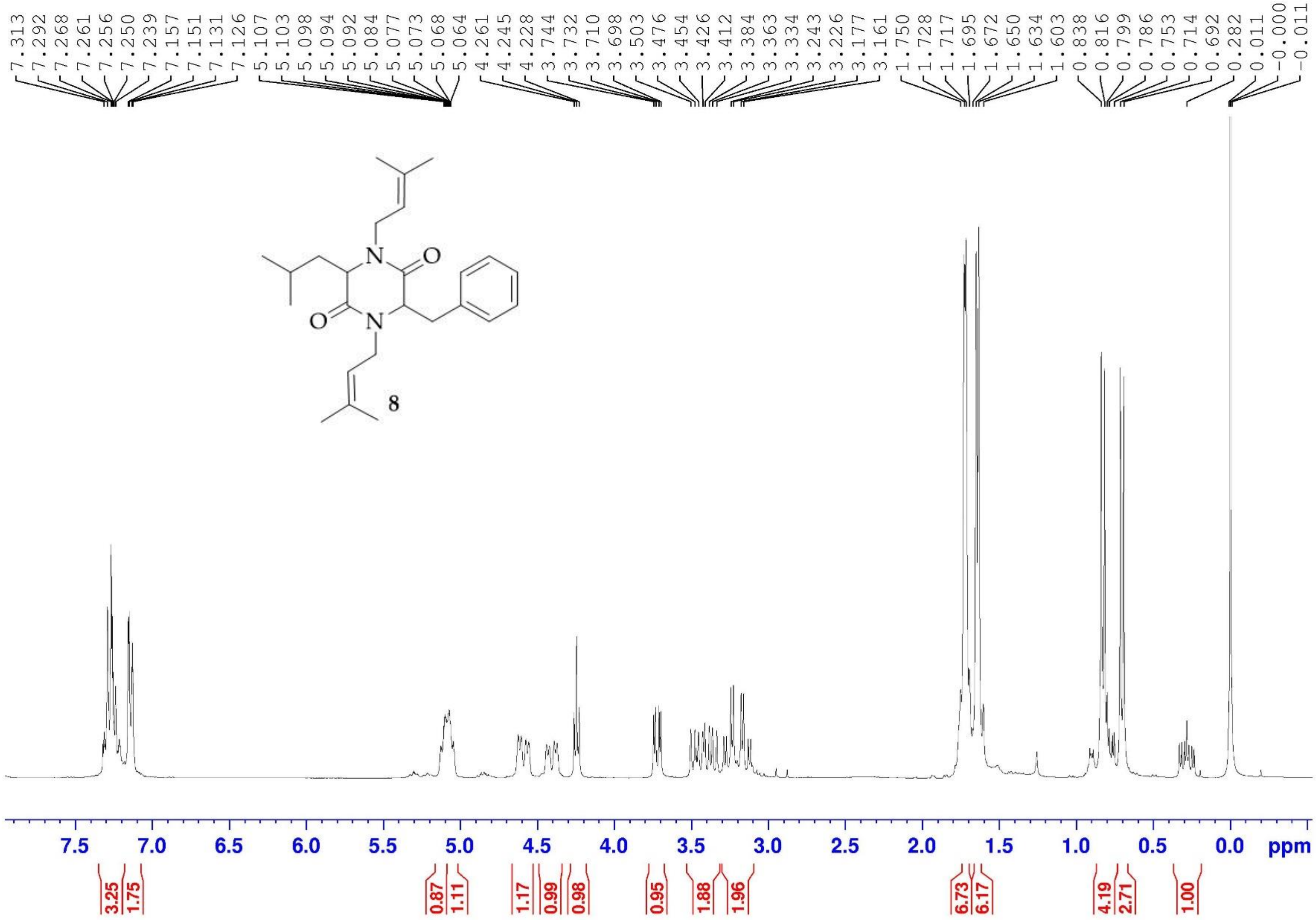

Figure S7. ^{1}H-NMR of the (3*S*,6*S*)-3-Benzyl-6-isobutyl-1,4-bis(3-methylbut-2-en-1-yl)-2,5-diketopiperazine (**8**).

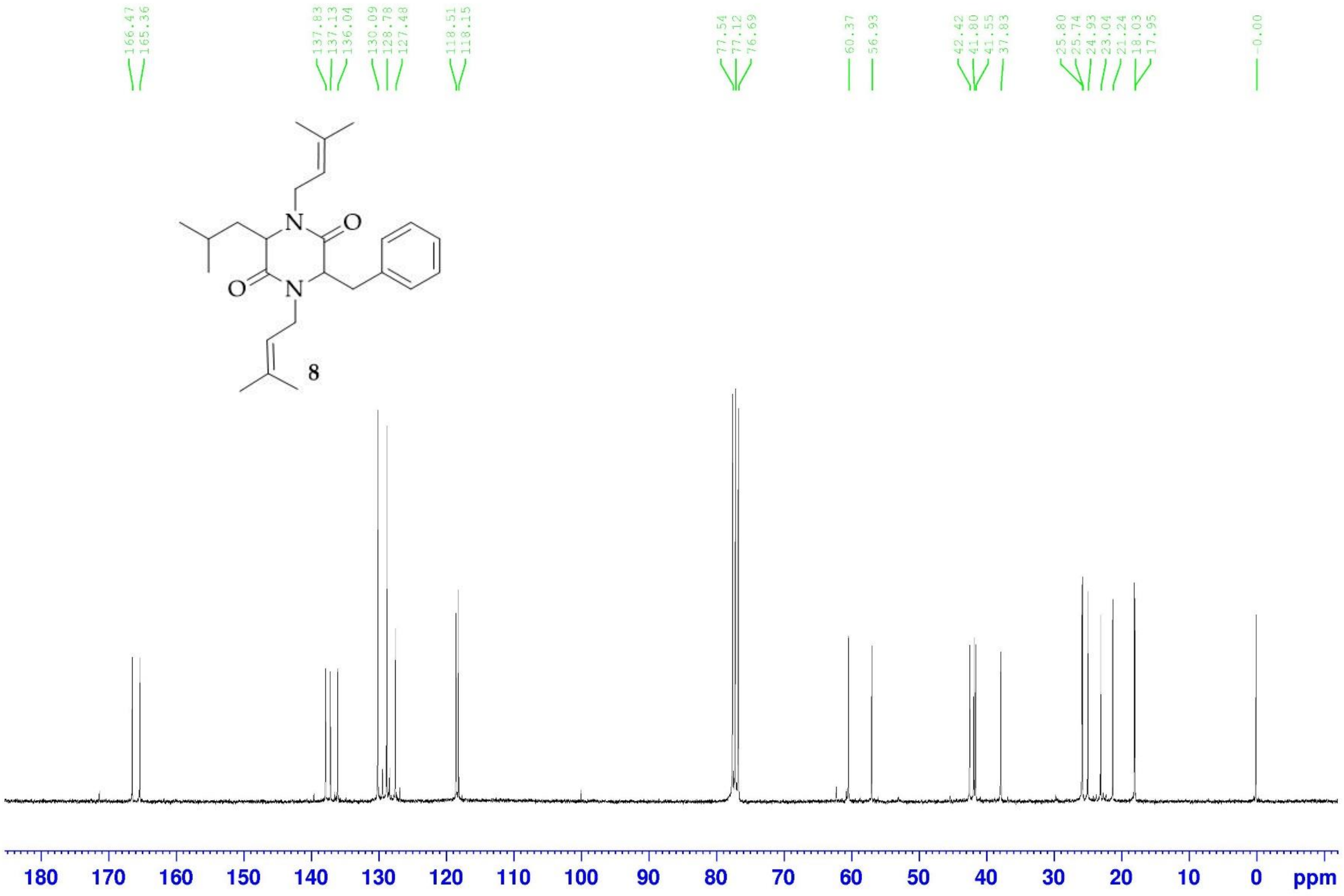

Figure S8. ^{13}C-NMR of the (*3S*,*6S*)-3-Benzyl-6-isobutyl-1,4-bis(3-methylbut-2-en-1-yl)-2,5-diketopiperazine (**8**).

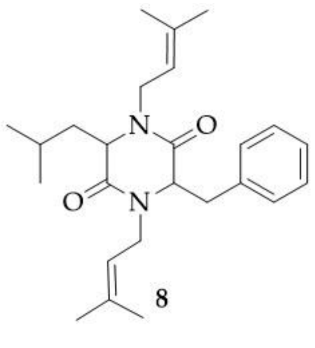

8

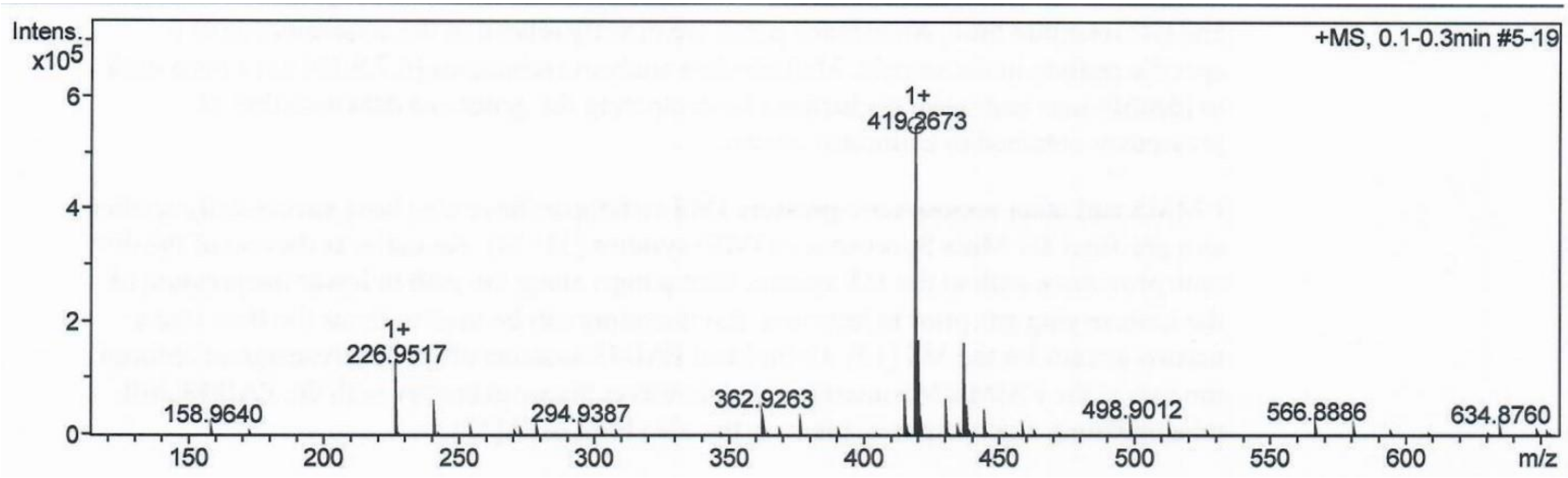

Figure S9. HRMS of the (*3S*,*6S*)-3-Benzyl-6-isobutyl-1,4-bis(3-methylbut-2-en-1-yl)-2,5-diketopiperazine (**8**).

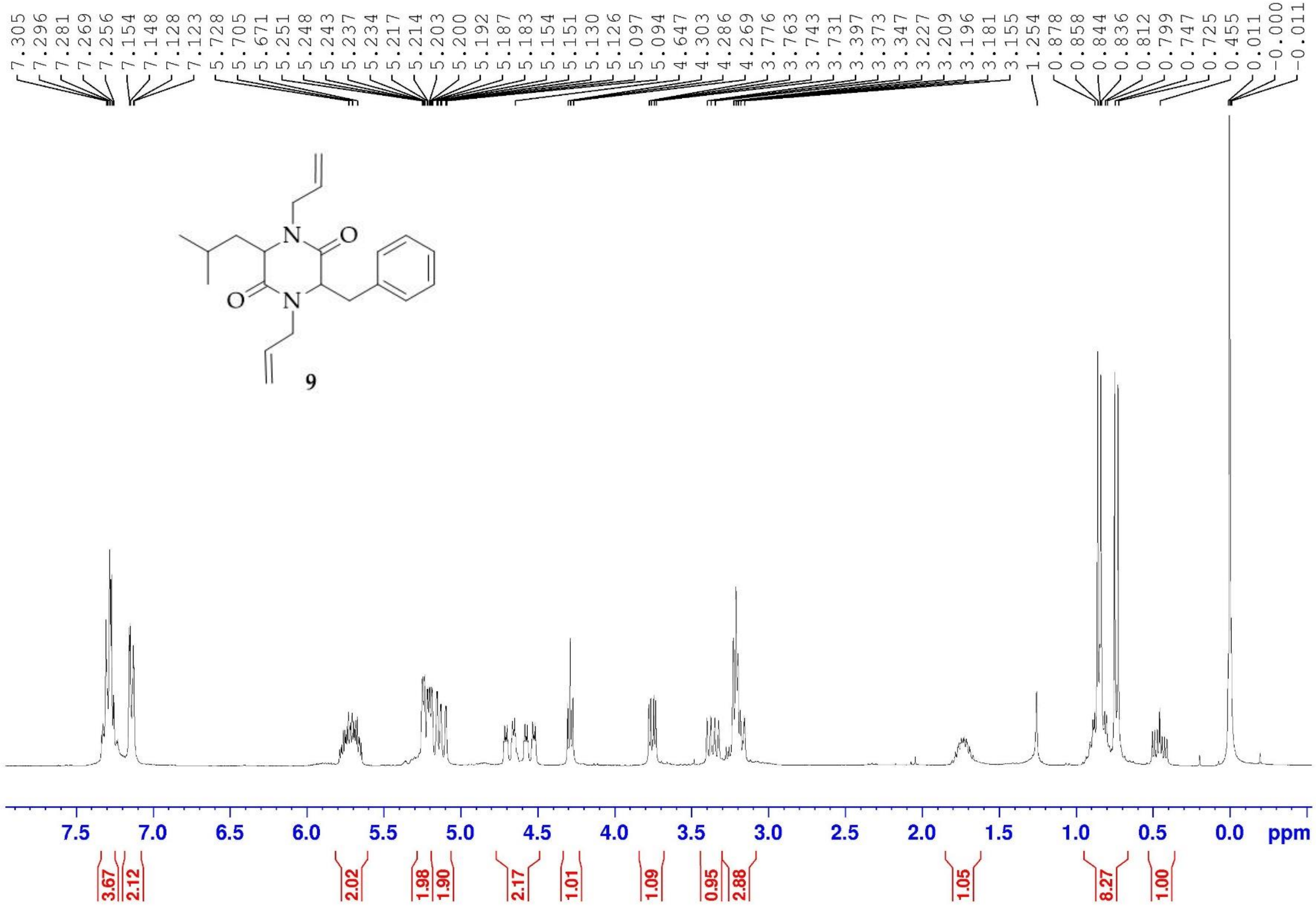

Figure S10. ^{1}H-NMR of the (3S,6S)-1,4-Diallyl-3-benzyl-6-isobutyl-2,5-diketopiperazine (**9**).

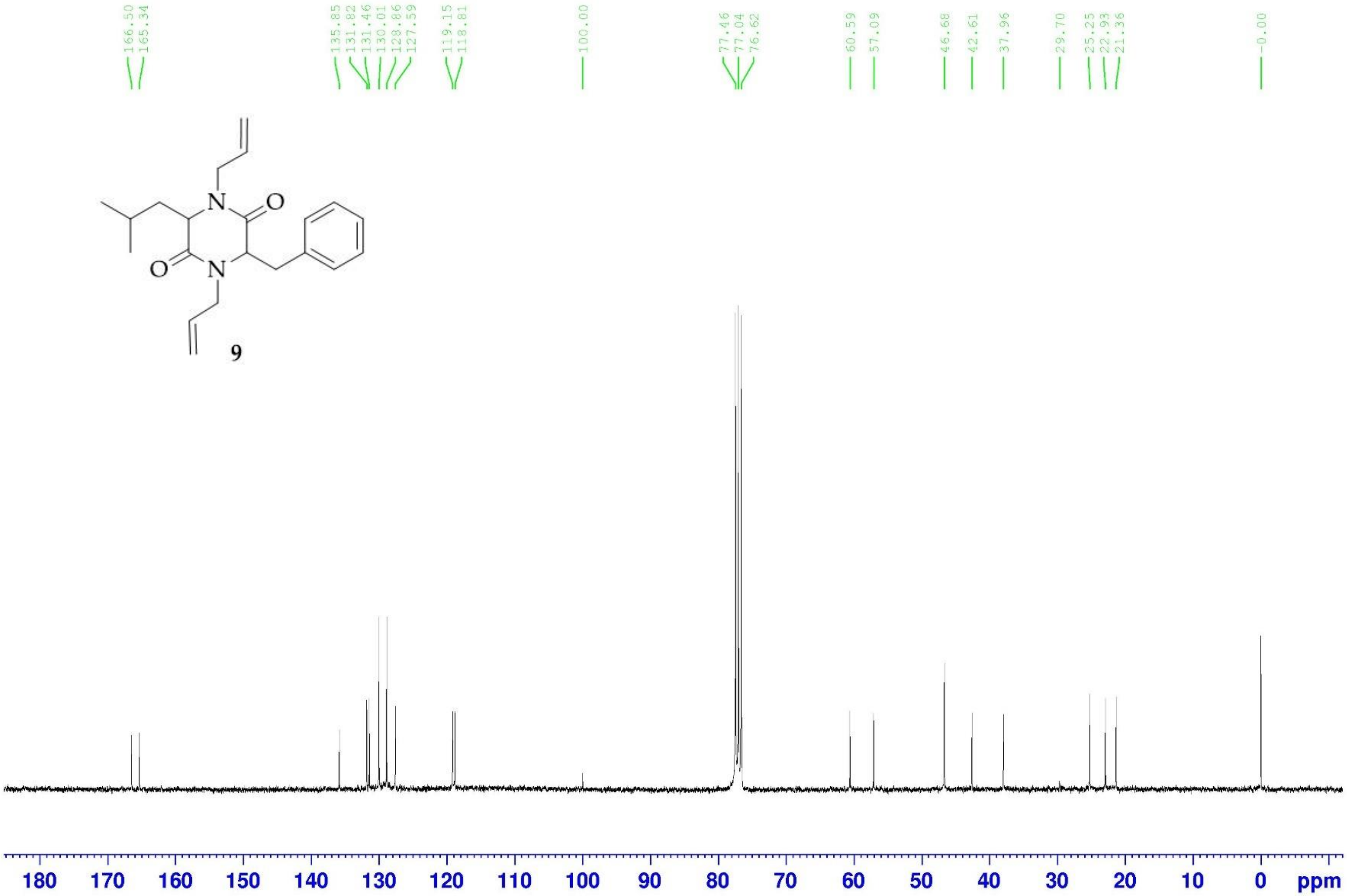

Figure S11. ^{13}C-NMR of the (3S,6S)-1,4-Diallyl-3-benzyl-6-isobutyl-2,5-diketopiperazine (**9**).

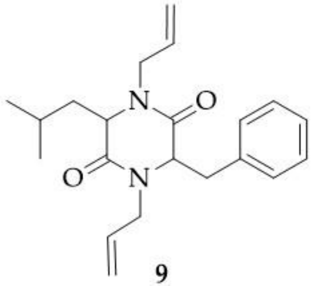

9

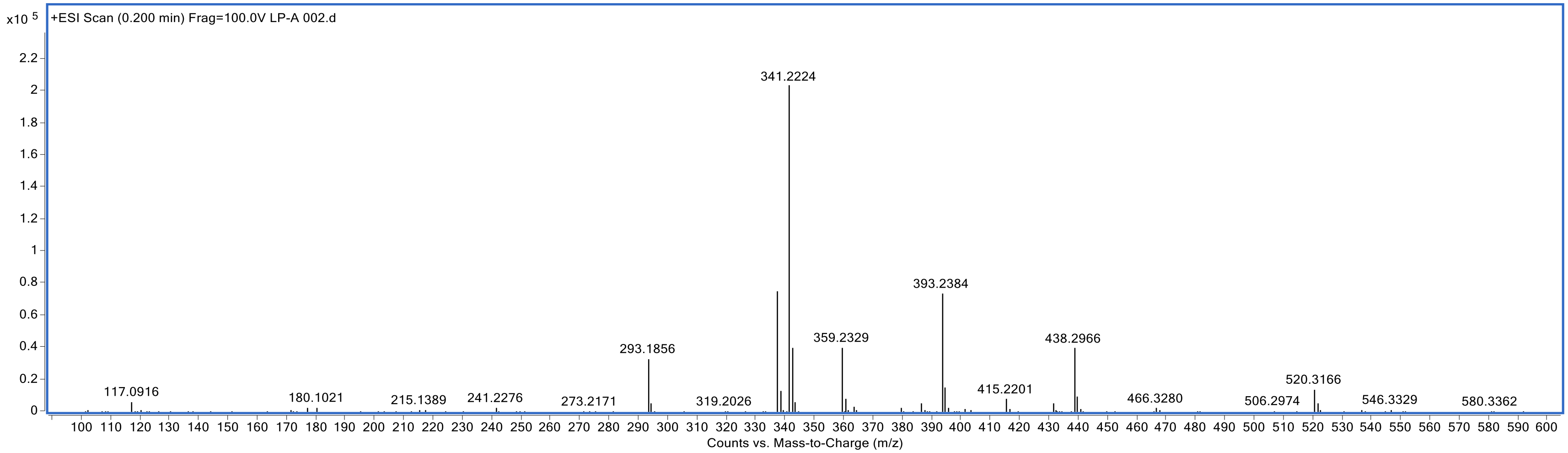

Figure S12. HRMS of the (3S,6S)-1,4-Diallyl-3-benzyl-6-isobutyl-2,5-diketopiperazine (**9**).

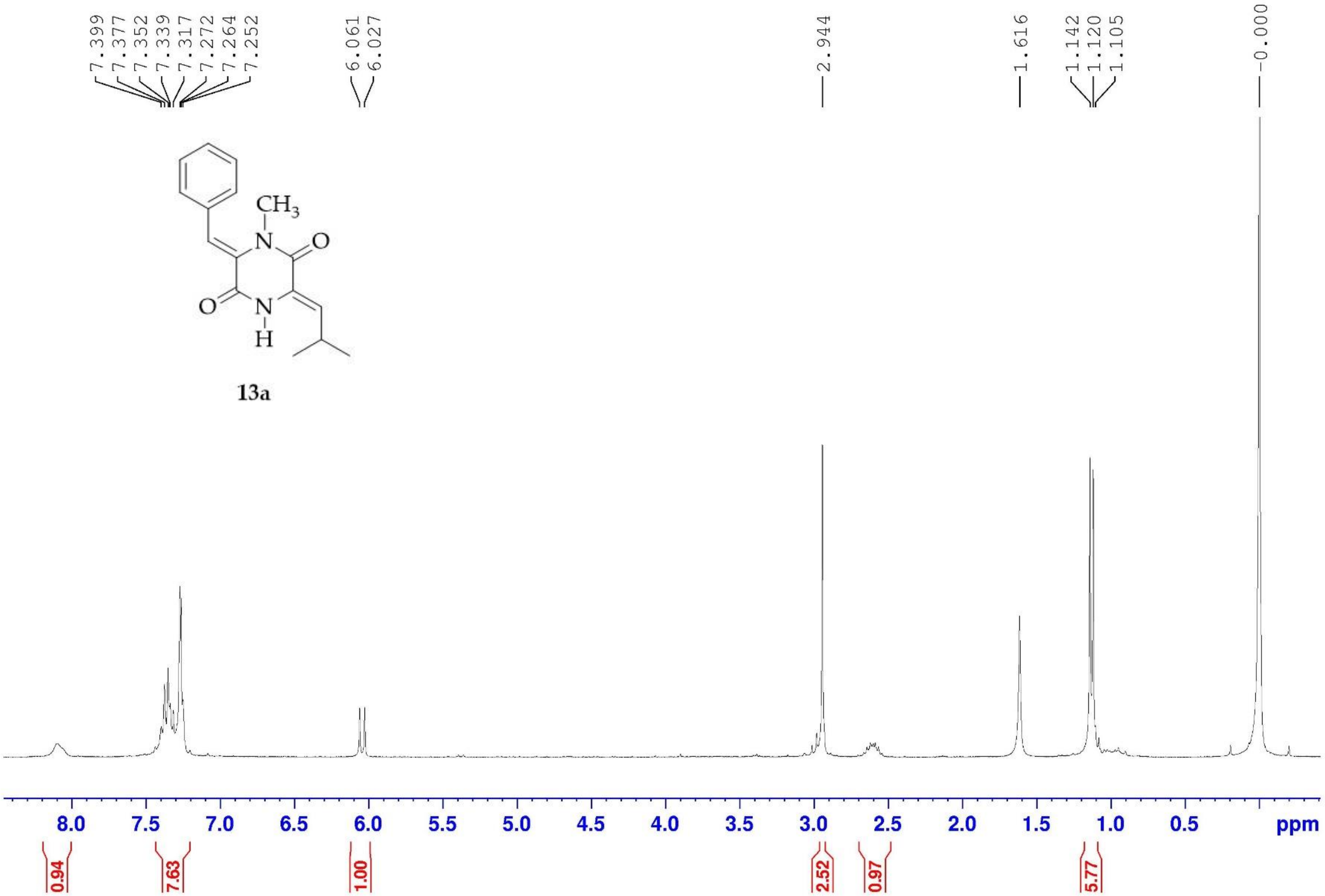

Figure S13. ^{1}H-NMR of the (*3Z,6Z*)-3-Benzylidene-6-(2-methylpropylidene)-4-methyl-2,5-diketopiperazine (**13a**).

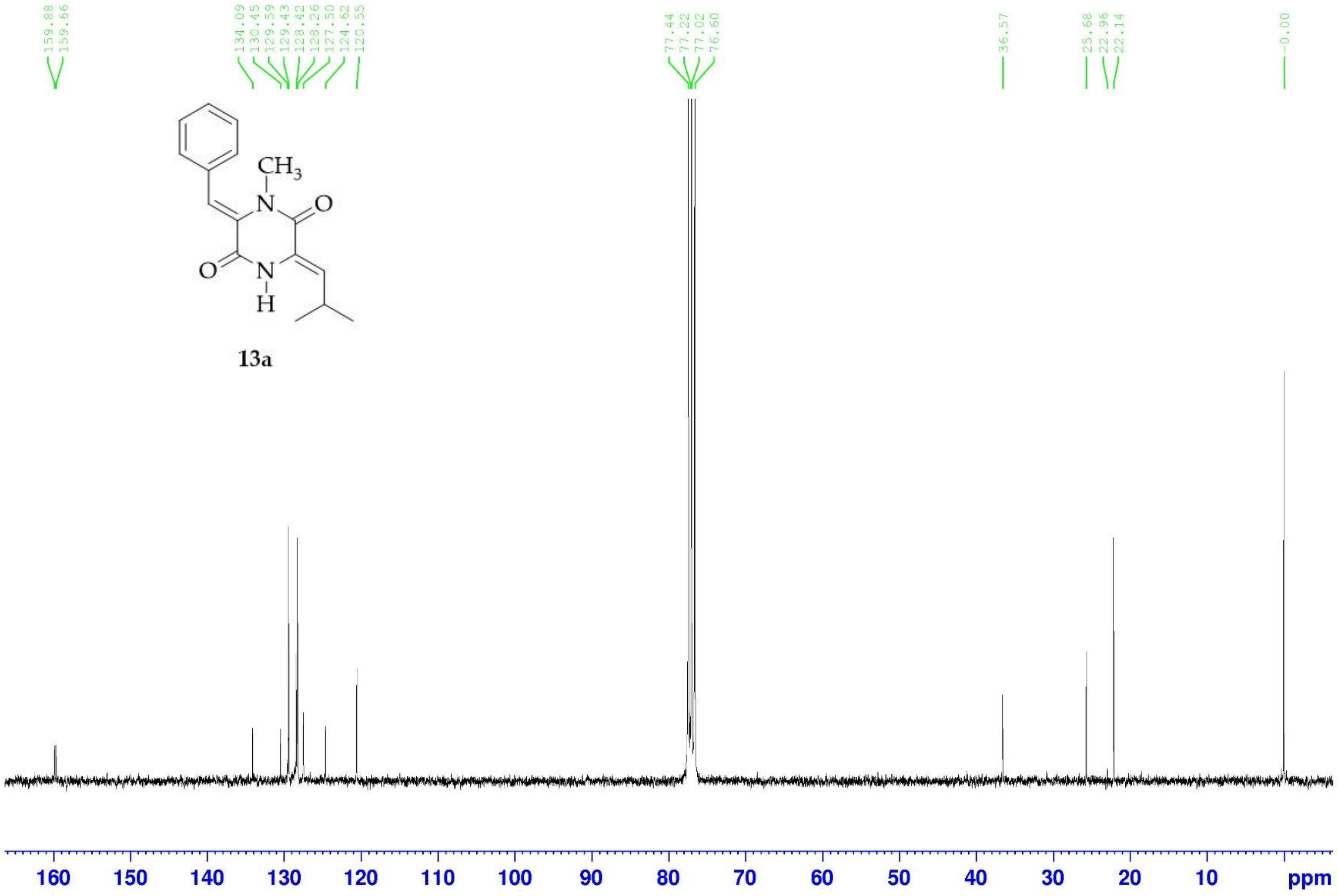

Figure S14. ^{13}C-NMR of the (*3Z,6Z*)-3-Benzylidene-6-(2-methylpropylidene)-4-methyl-2,5-diketopiperazine (**13a**).

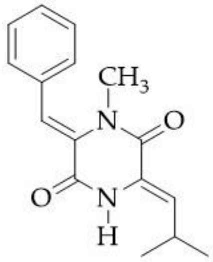

13a

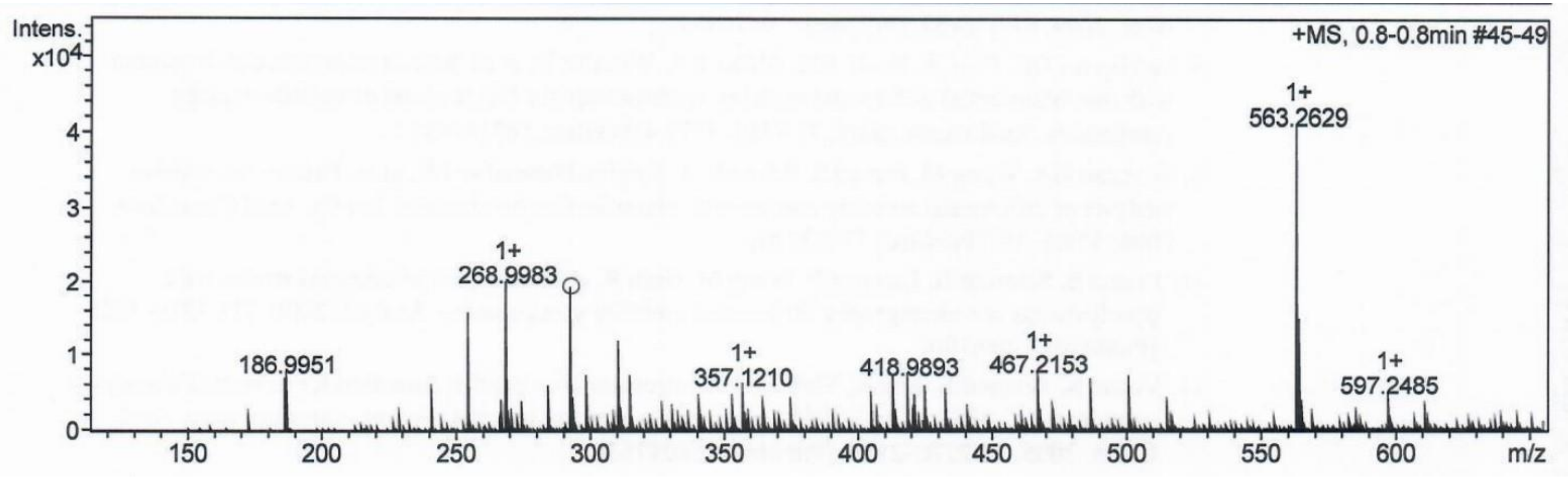

Figure S15. HRMS of the (*3Z,6Z*)-3-Benzylidene-6-(2-methylpropylidene)-4-methyl-2,5-diketopiperazine (**13a**).

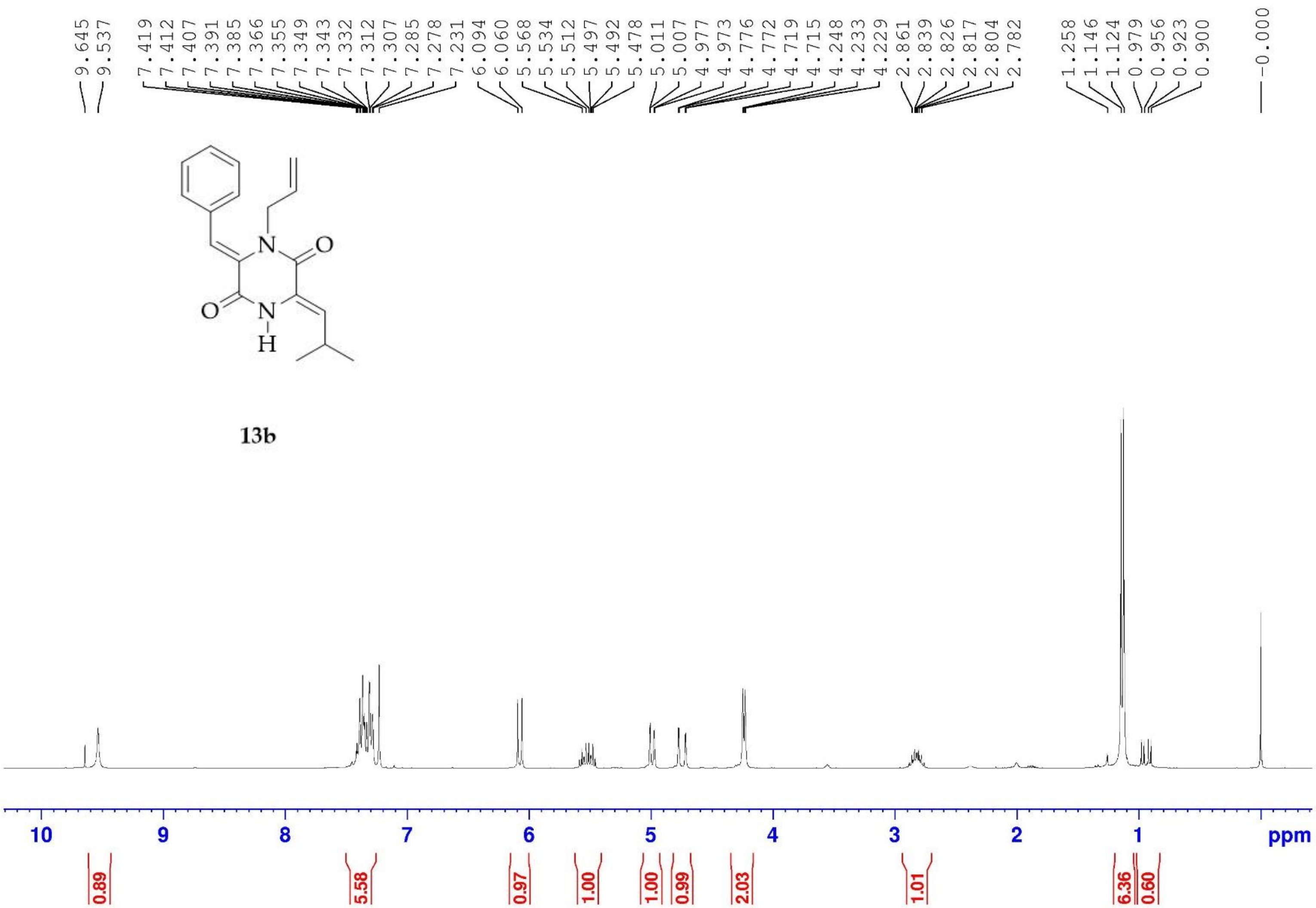

Figure S16. ^{1}H-NMR of the (3*Z*,6*Z*)-4-Allyl-3-benzylidene-6-(2-methylpropylidene)-2,5-diketopiperazine (**13b**).

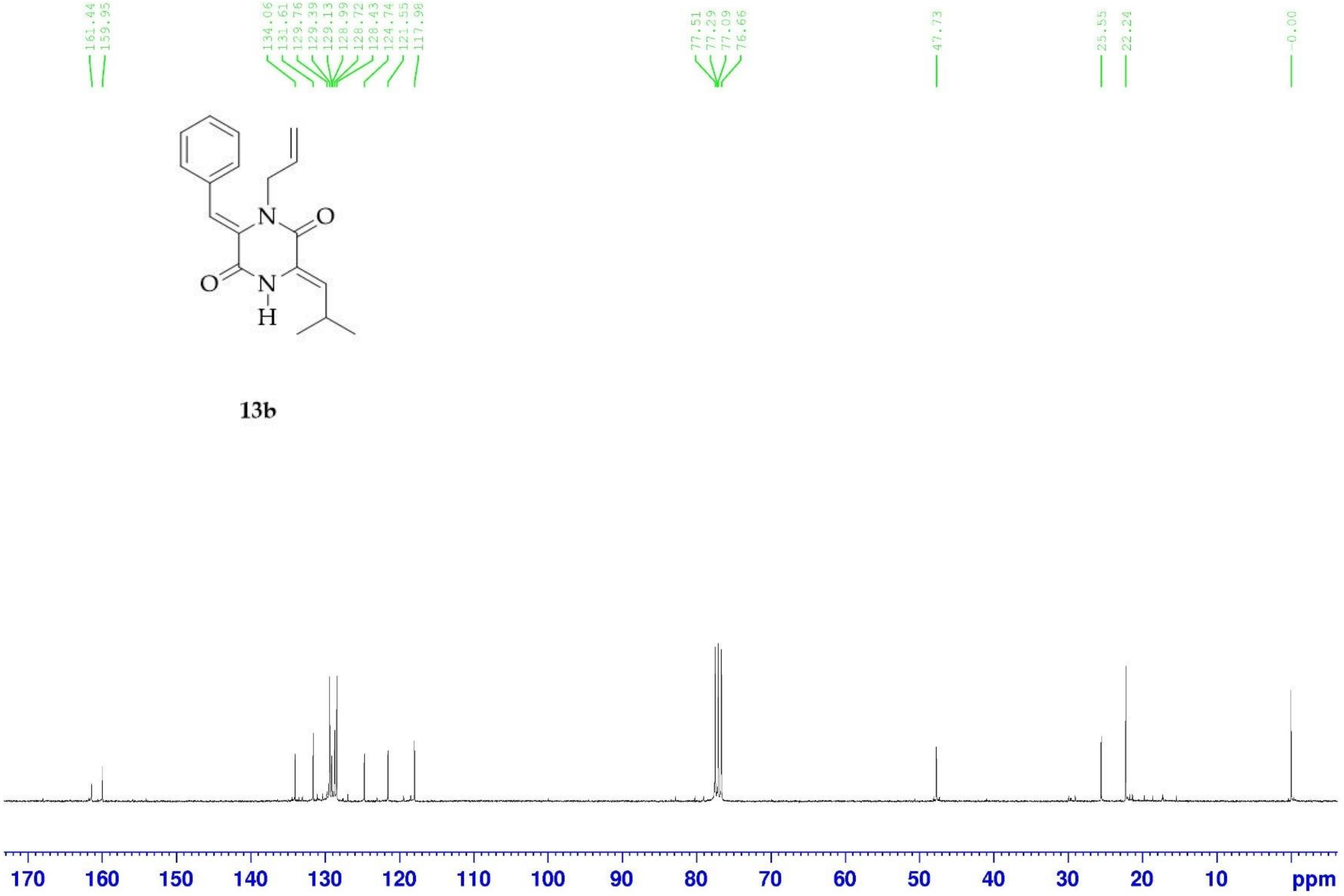

Figure S17. ^{13}C-NMR of the (*3Z,6Z*)-4-Allyl-3-benzylidene-6-(2-methylpropylidene)-2,5-diketopiperazine (**13b**).

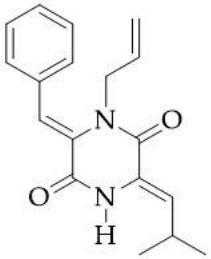

13b

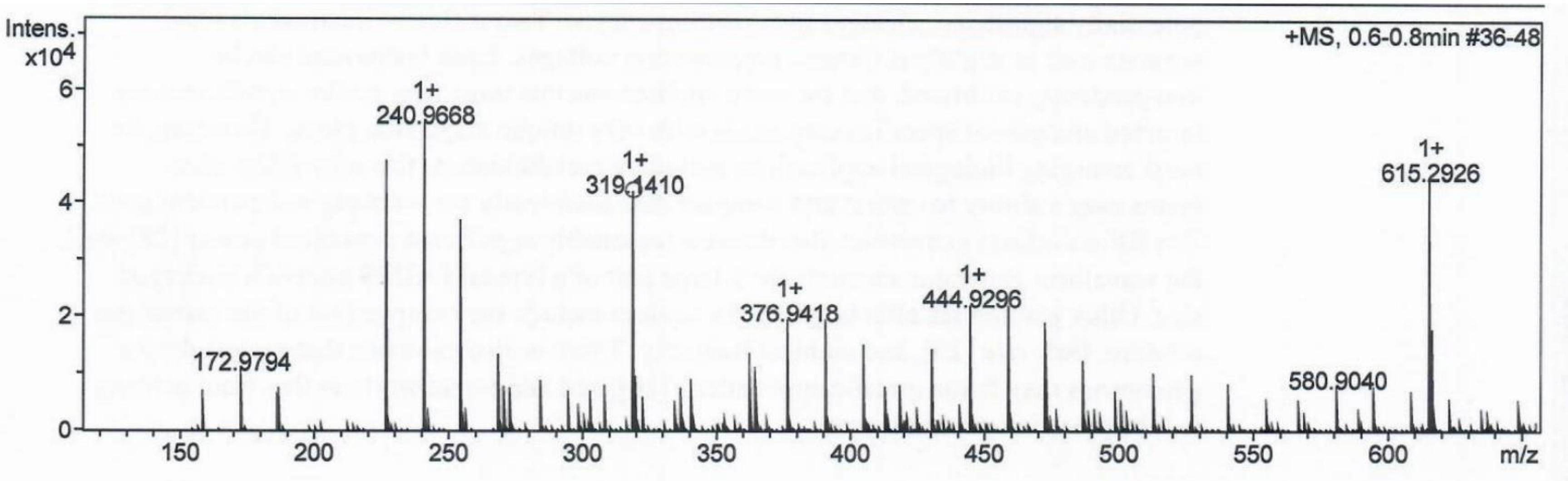

Figure S18. HRMS of the (*3Z,6Z*)-4-Allyl-3-benzylidene-6-(2-methylpropylidene)-2,5-diketopiperazine (**13b**).

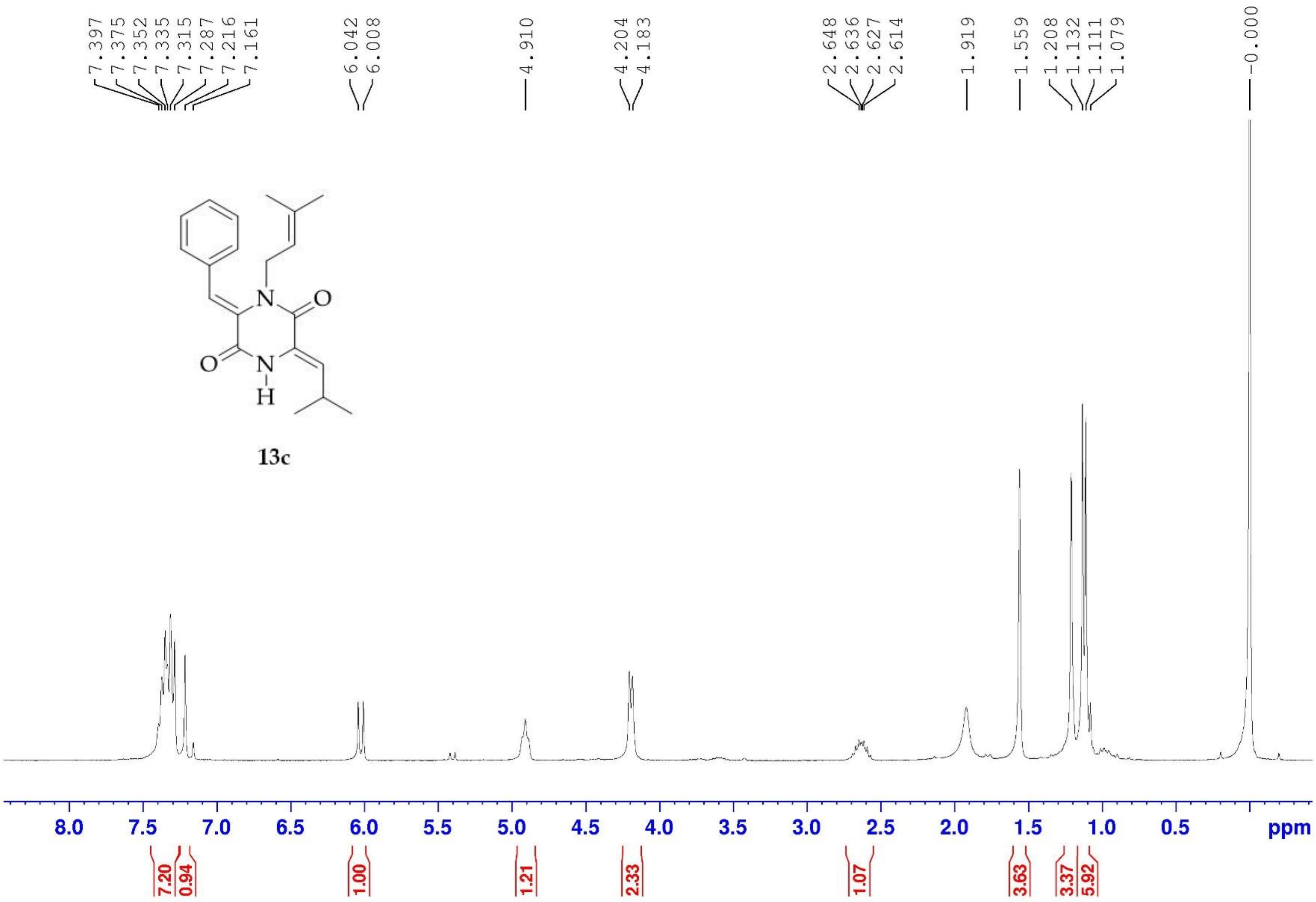

Figure S19. ^{1}H-NMR of the (*3Z,6Z*)-3-Benzylidene-6-(2-methylpropylidene)-4-(3-methyl but-2-en-1-yl)-2,5-diketopiperazine (**13c**).

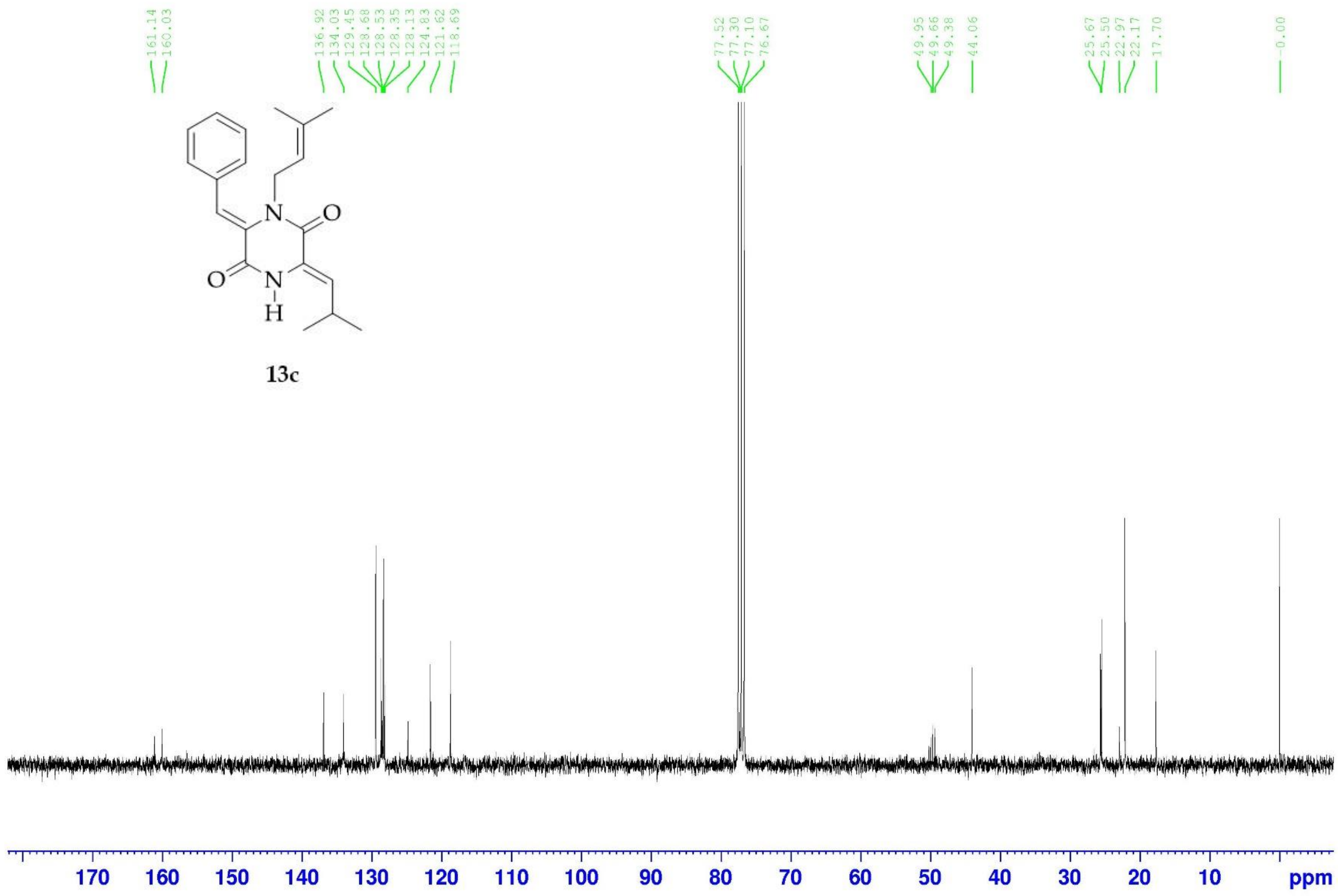

Figure S20. ^{13}C-NMR of the (3*Z*,6*Z*)-3-Benzylidene-6-(2-methylpropylidene)-4-(3-methyl but-2-en-1-yl)-2,5-diketopiperazine (**13c**).

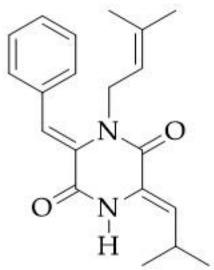

13c

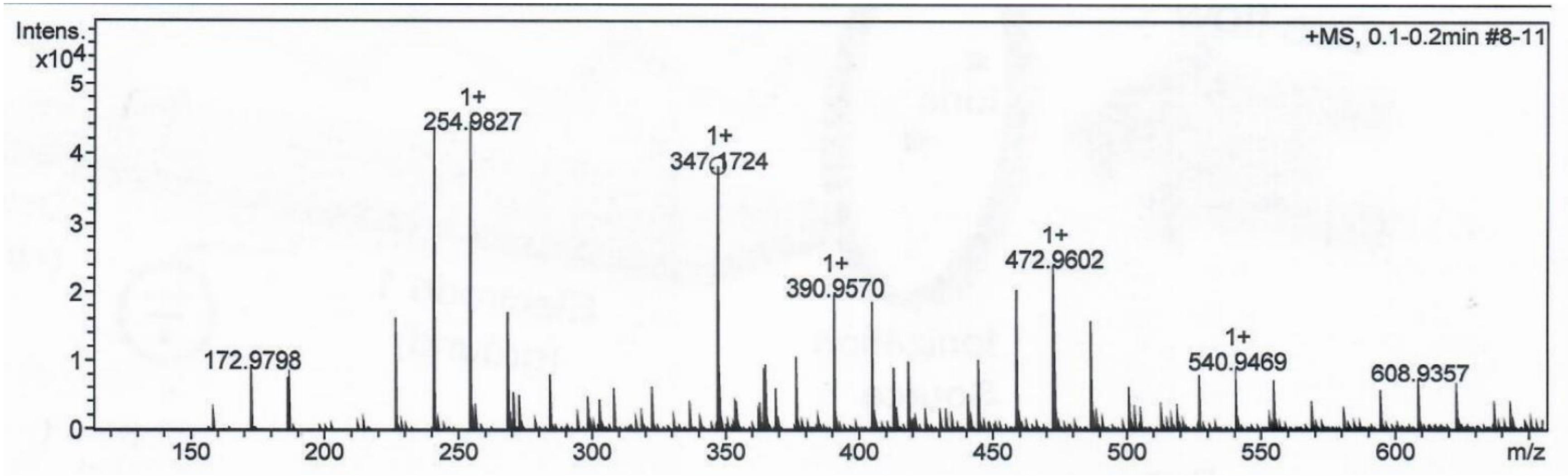

Figure S21. HRMS of the (*3Z*,*6Z*)-3-Benzylidene-6-(2-methylpropylidene)-4-(3-methyl but-2-en-1-yl)-2,5-diketopiperazine (**13c**).

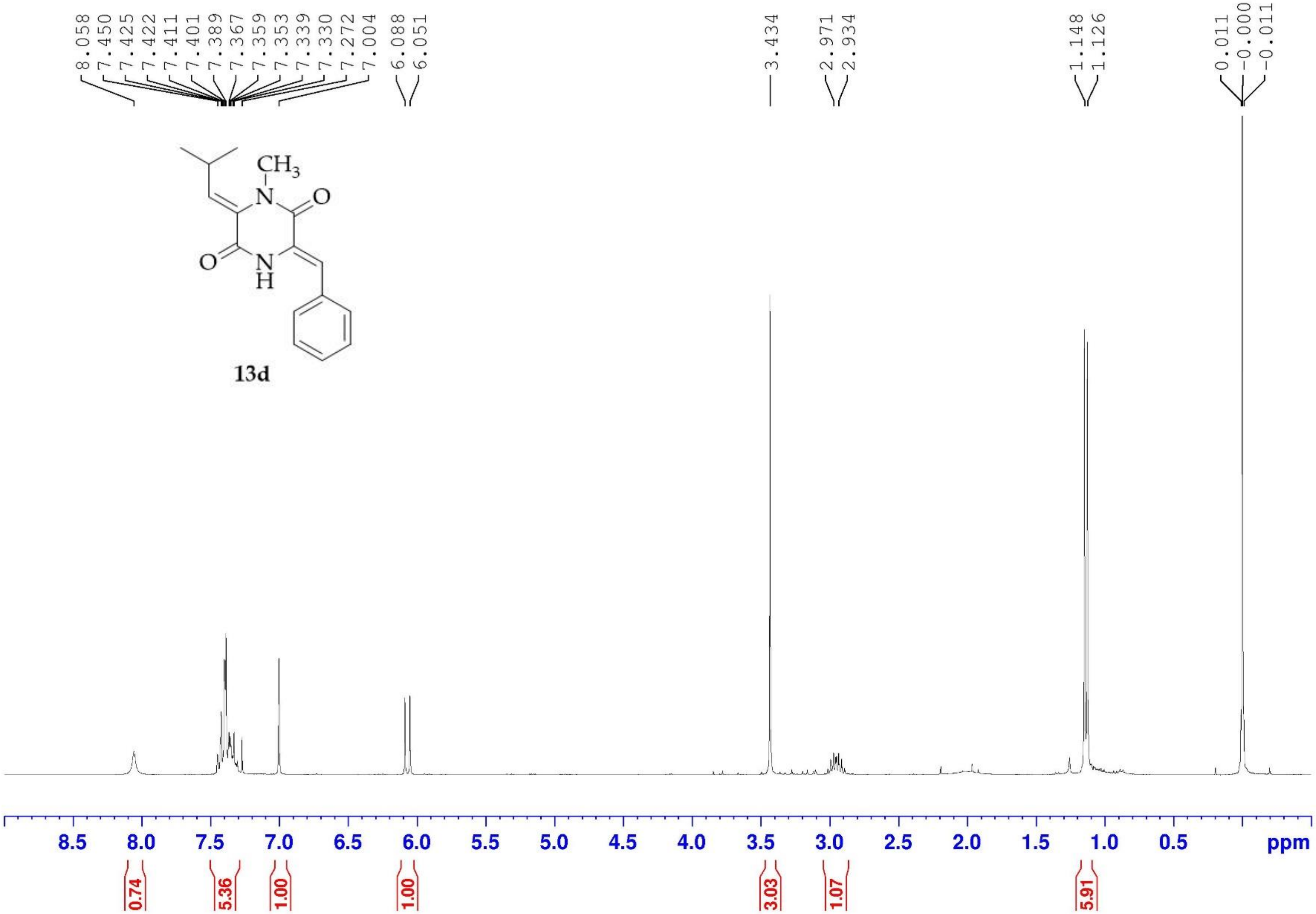

Figure S22. ^{1}H-NMR of the (3*Z*,6*Z*)-3-Benzylidene-6-(2-methylpropylidene)-1-methyl-2,5-diketopiperazine (**13d**).

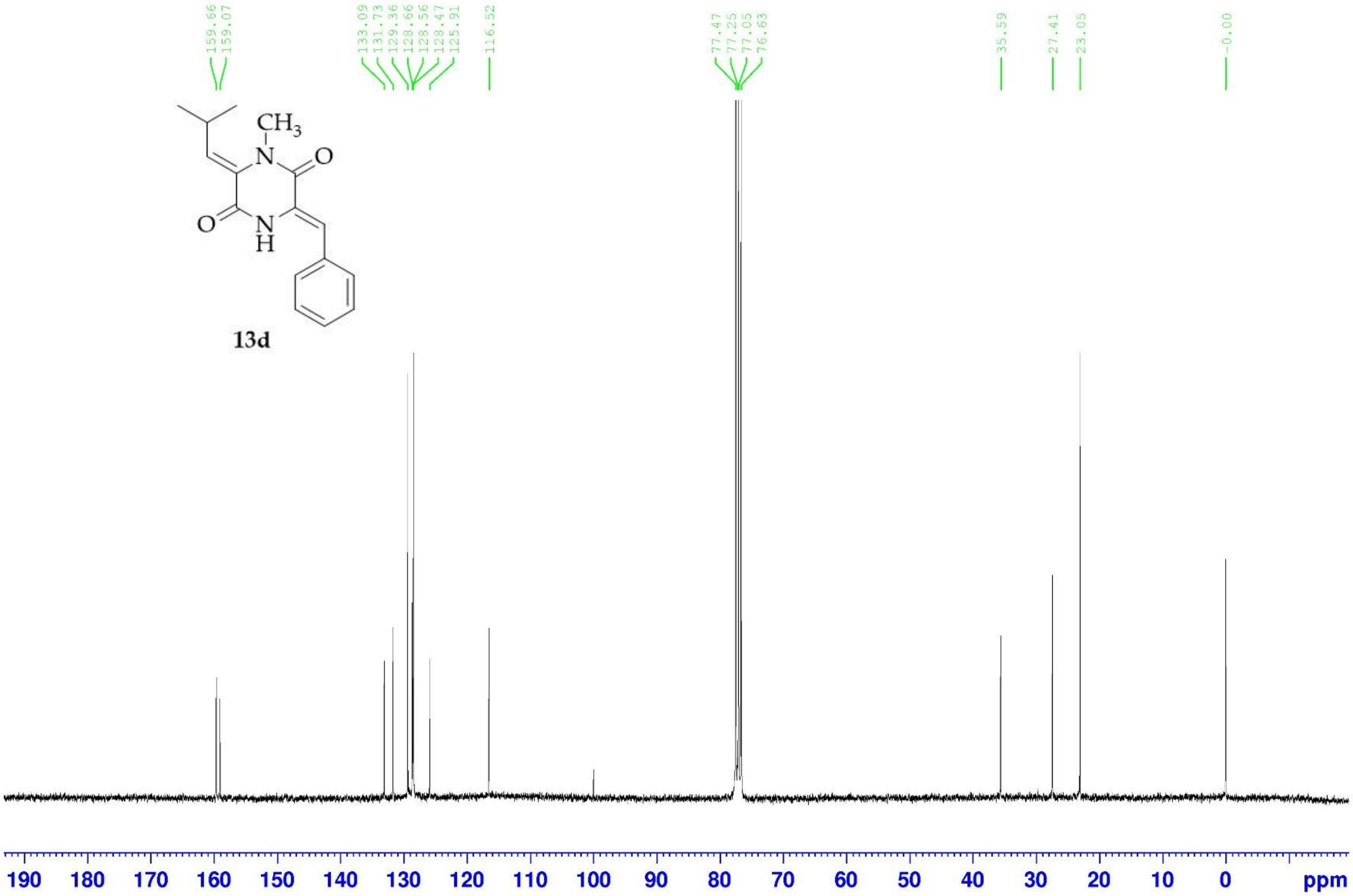

Figure S23. ^{13}C-NMR of the (*3Z,6Z*)-3-Benzylidene-6-(2-methylpropylidene)-1-methyl-2,5-diketopiperazine (**13d**).

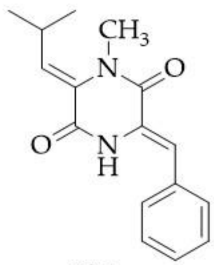

13d

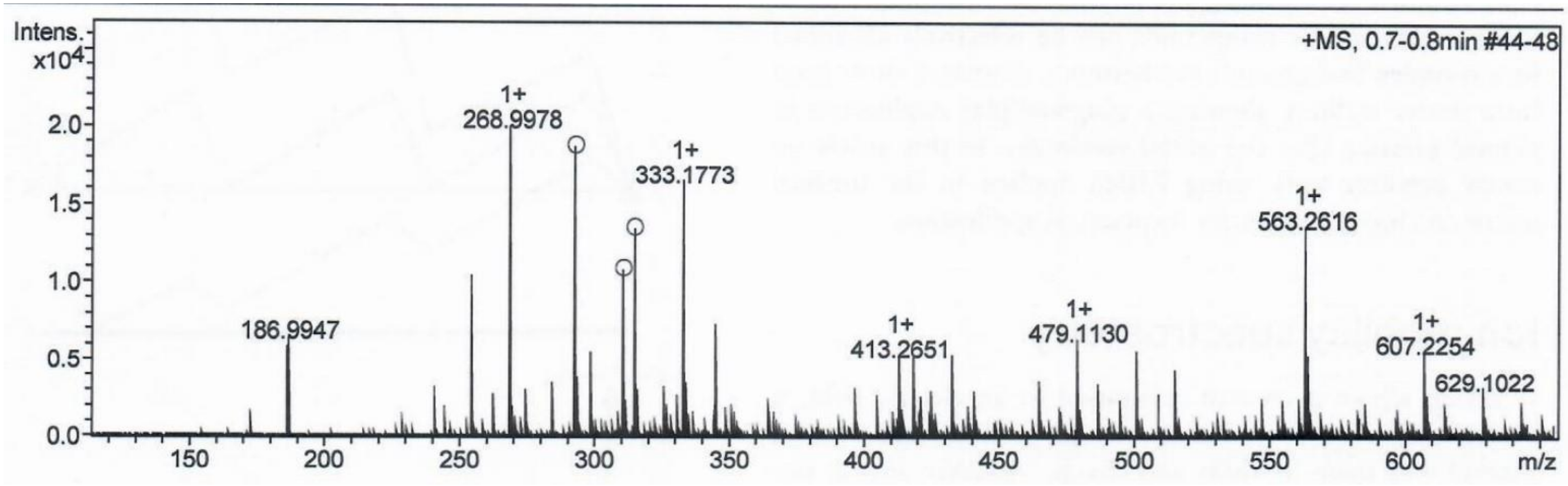

Figure S24. HRMS of the (*3Z,6Z*)-3-Benzylidene-6-(2-methylpropylidene)-1-methyl-2,5-diketopiperazine (**13d**).

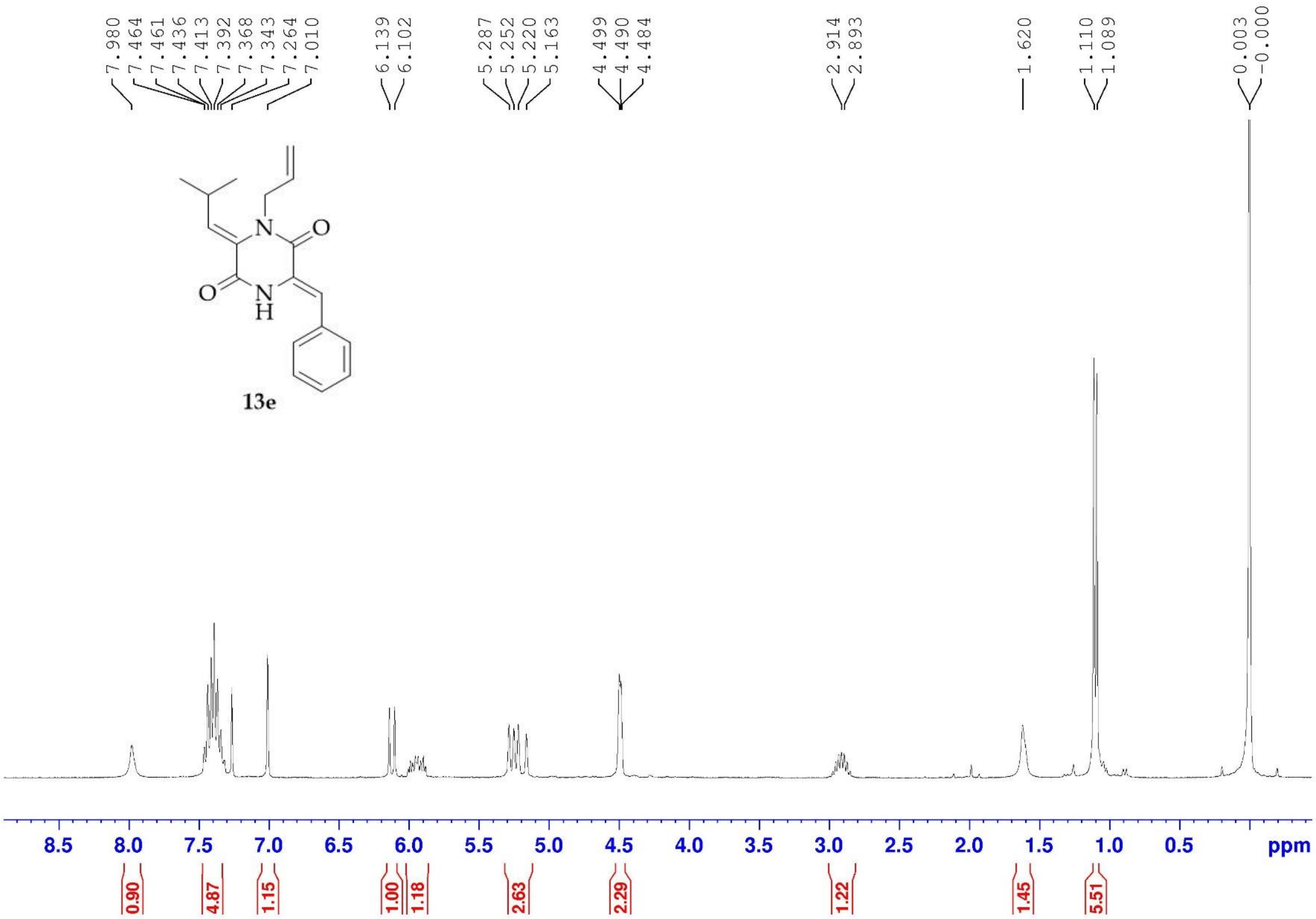

Figure S25. ^{1}H-NMR of the (*3Z*,*6Z*)-1-Allyl-3-benzylidene-6-(2-methylpropylidene)-2,5-diketopiperazine (**13e**).

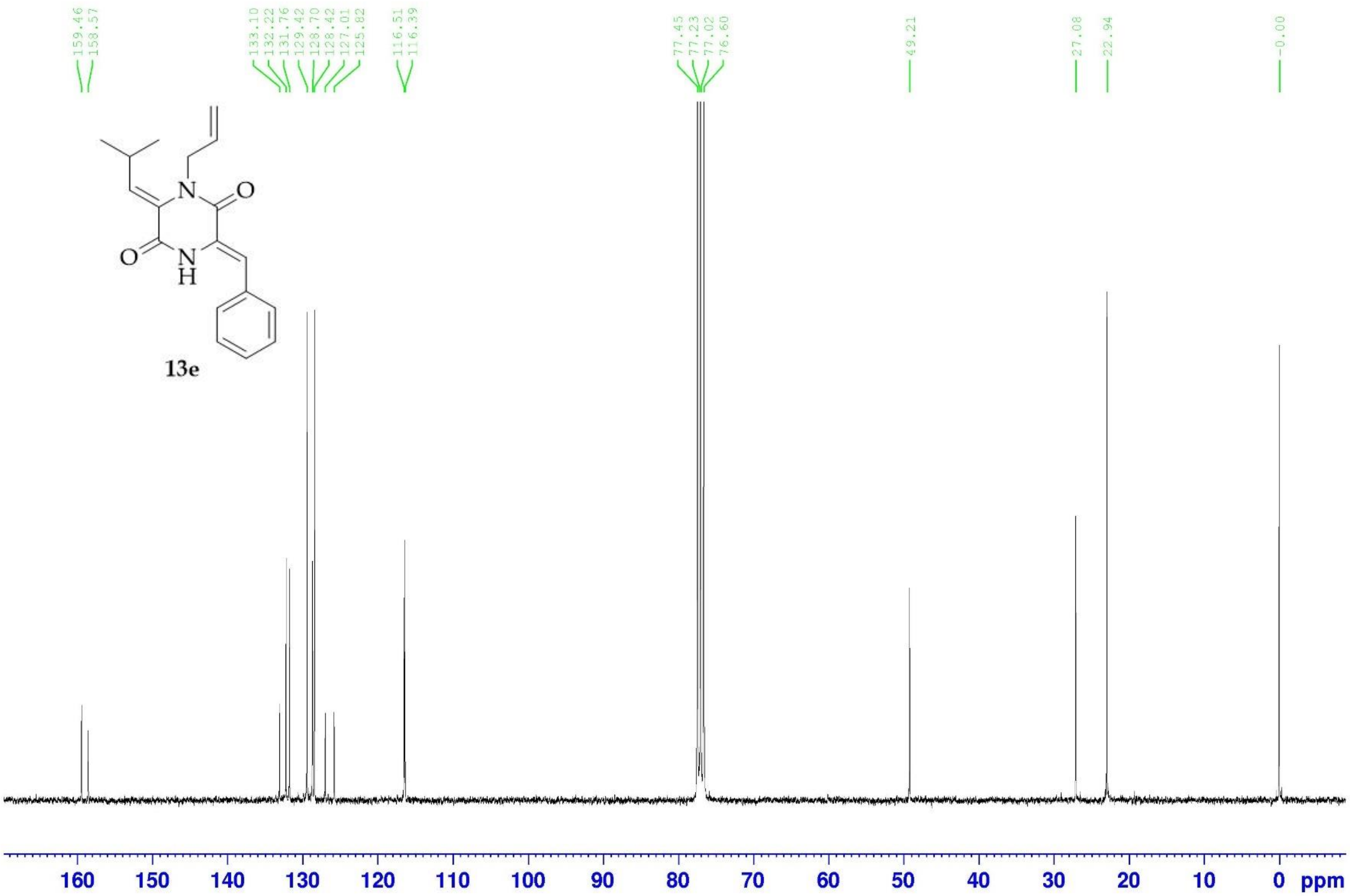

Figure S26. ^{13}C-NMR of the (3*Z*,6*Z*)-1-Allyl-3-benzylidene-6-(2-methylpropylidene)-2,5-diketopiperazine (**13e**).

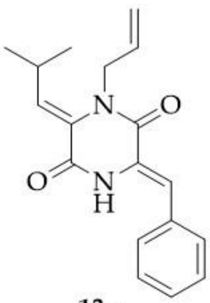

13e

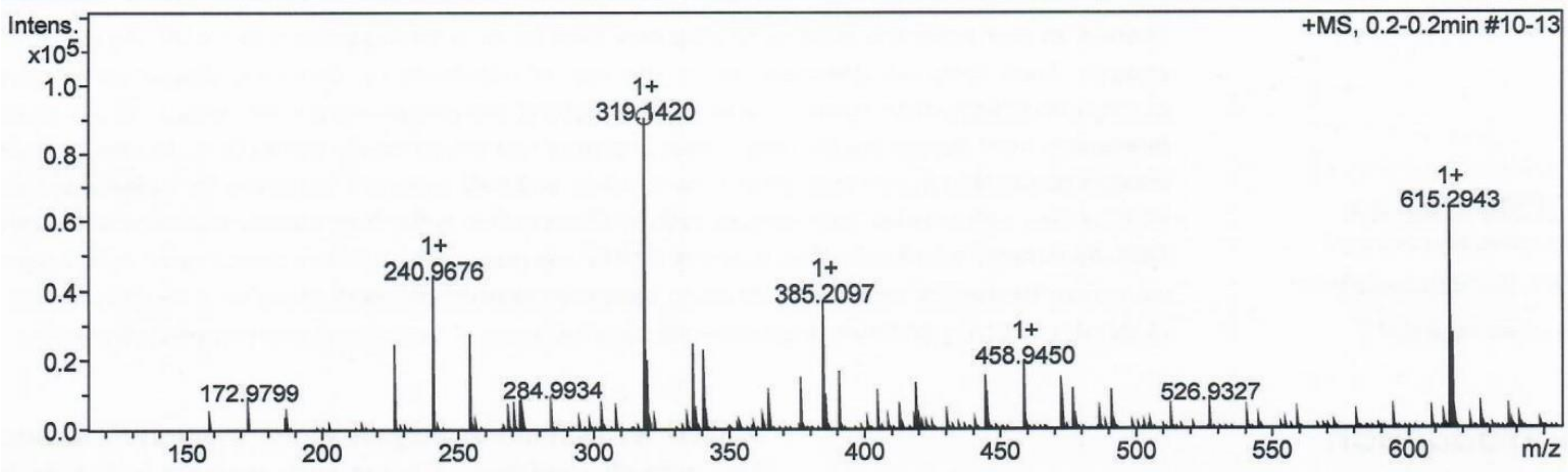

Figure S27. HRMS of the (*3Z,6Z*)-1-Allyl-3-benzylidene-6-(2-methylpropylidene)-2,5-diketopiperazine (**13e**).

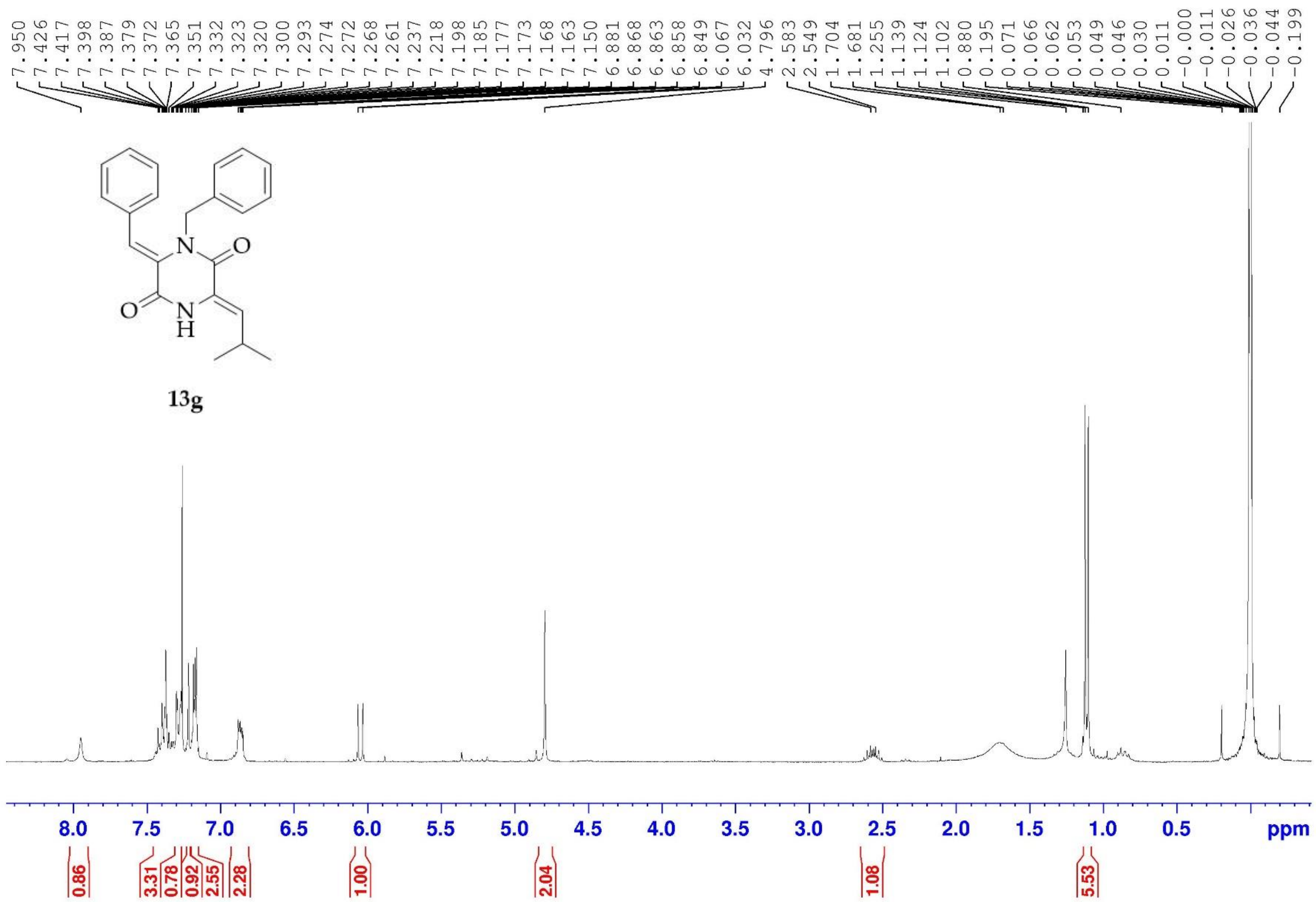

13g

Figure S28. ^{1}H-NMR of the (*3Z,6Z*)-4-Benzyl-3-benzylidene-6-(2-methylpropylidene)-2,5-diketopiperazine (**13g**).

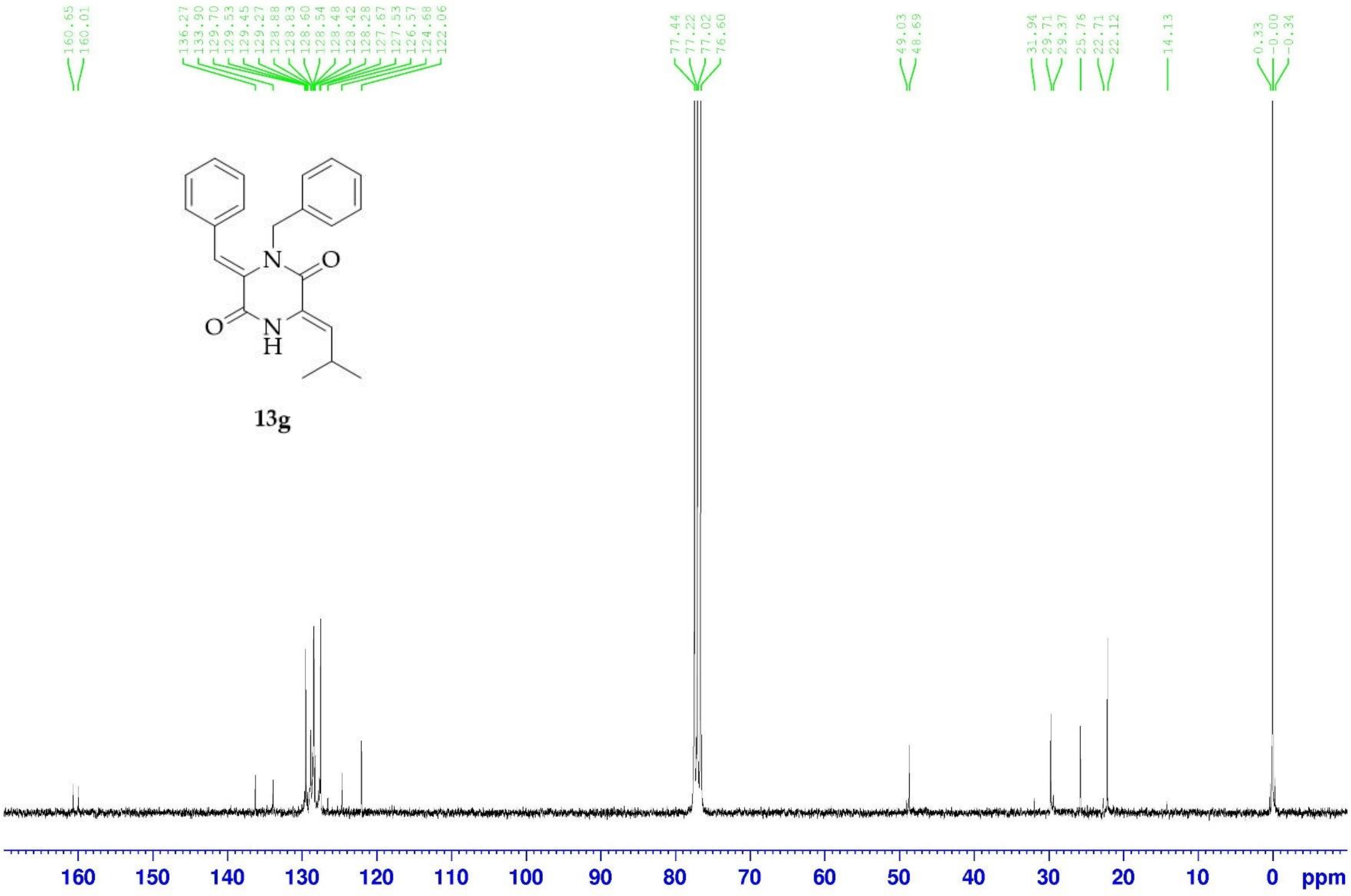

Figure S29. ^{13}C-NMR of the (*3Z,6Z*)-4-Benzyl-3-benzylidene-6-(2-methylpropylidene)-2,5-diketopiperazine (**13g**).

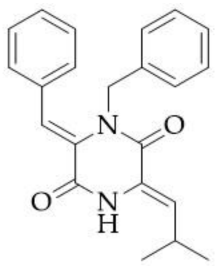

13g

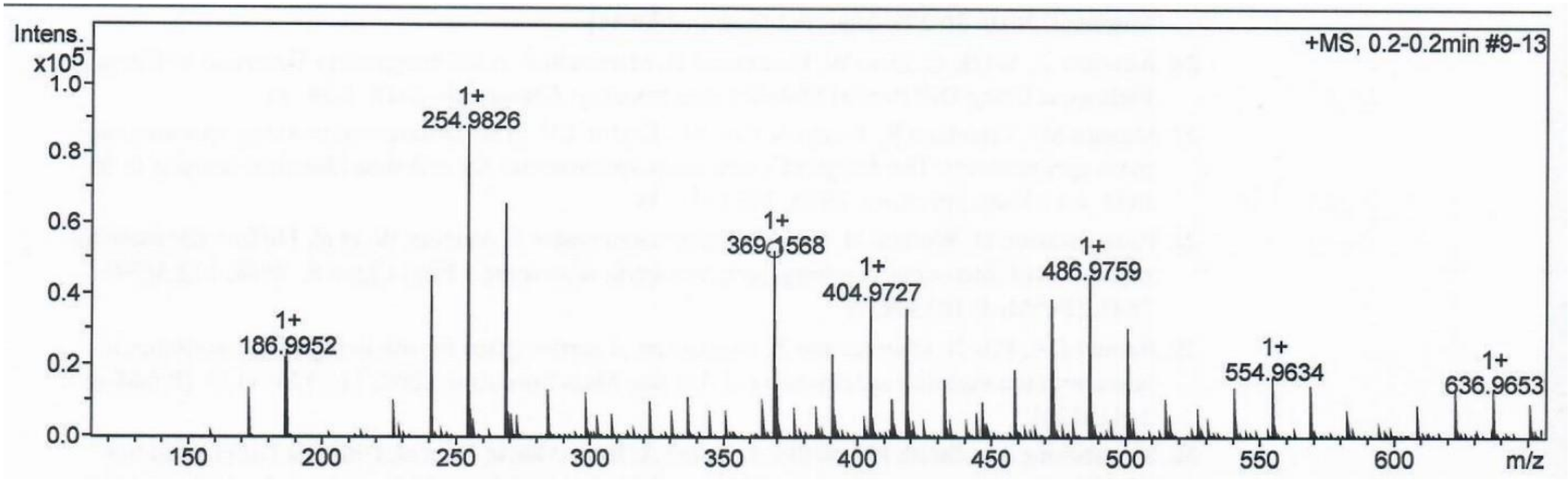

Figure S30. HRMS of the (*3Z,6Z*)-4-Benzyl-3-benzylidene-6-(2-methylpropylidene)-2,5-diketopiperazine (**13g**).

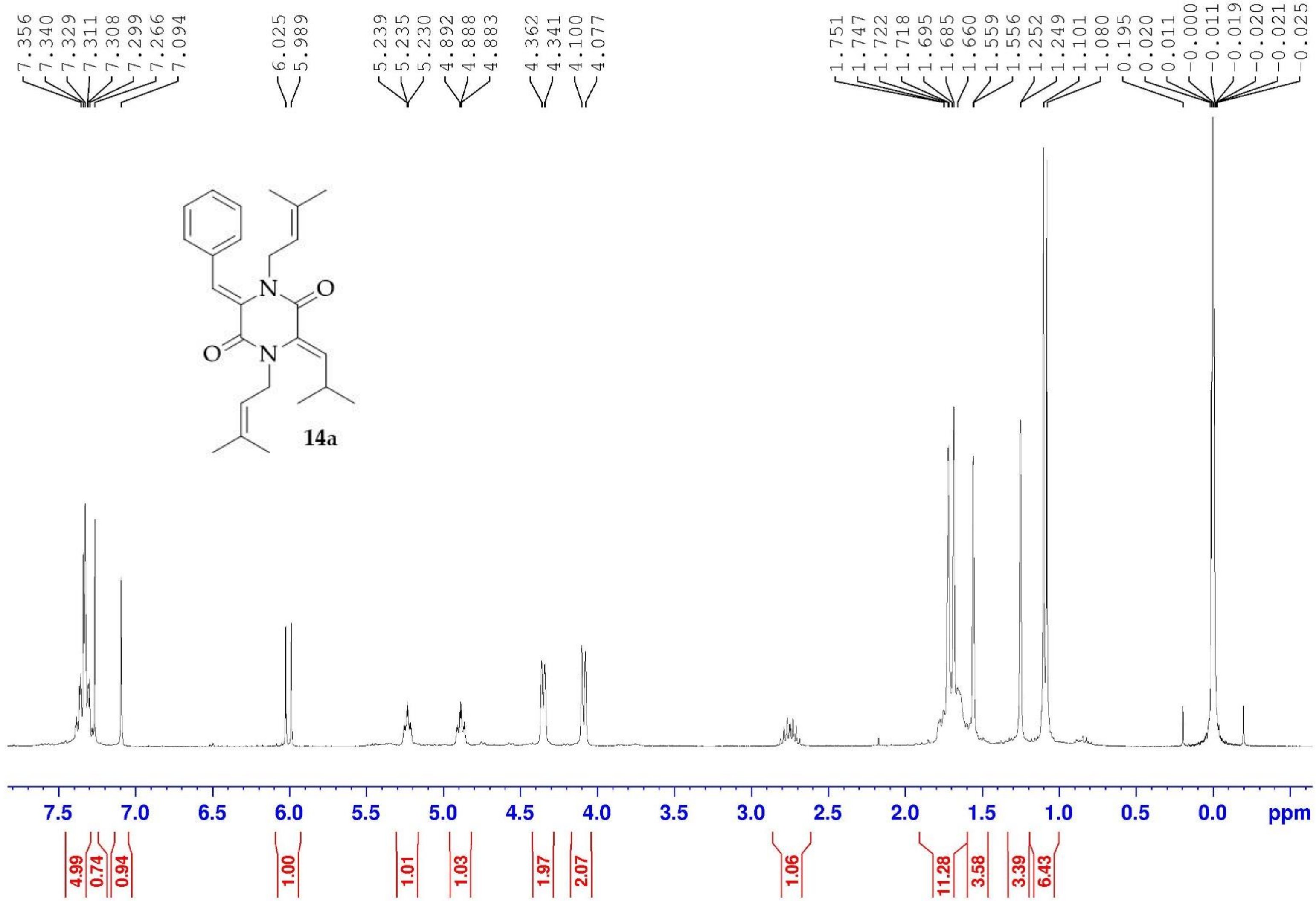

Figure S31. ^{1}H-NMR of the (*3Z,6Z*)-3-Benzylidene-6-(2-methylpropylidene)-1,4-bis(-3-methylbut-2-en-1yl)-2,5-diketopiperazine (**14a**).

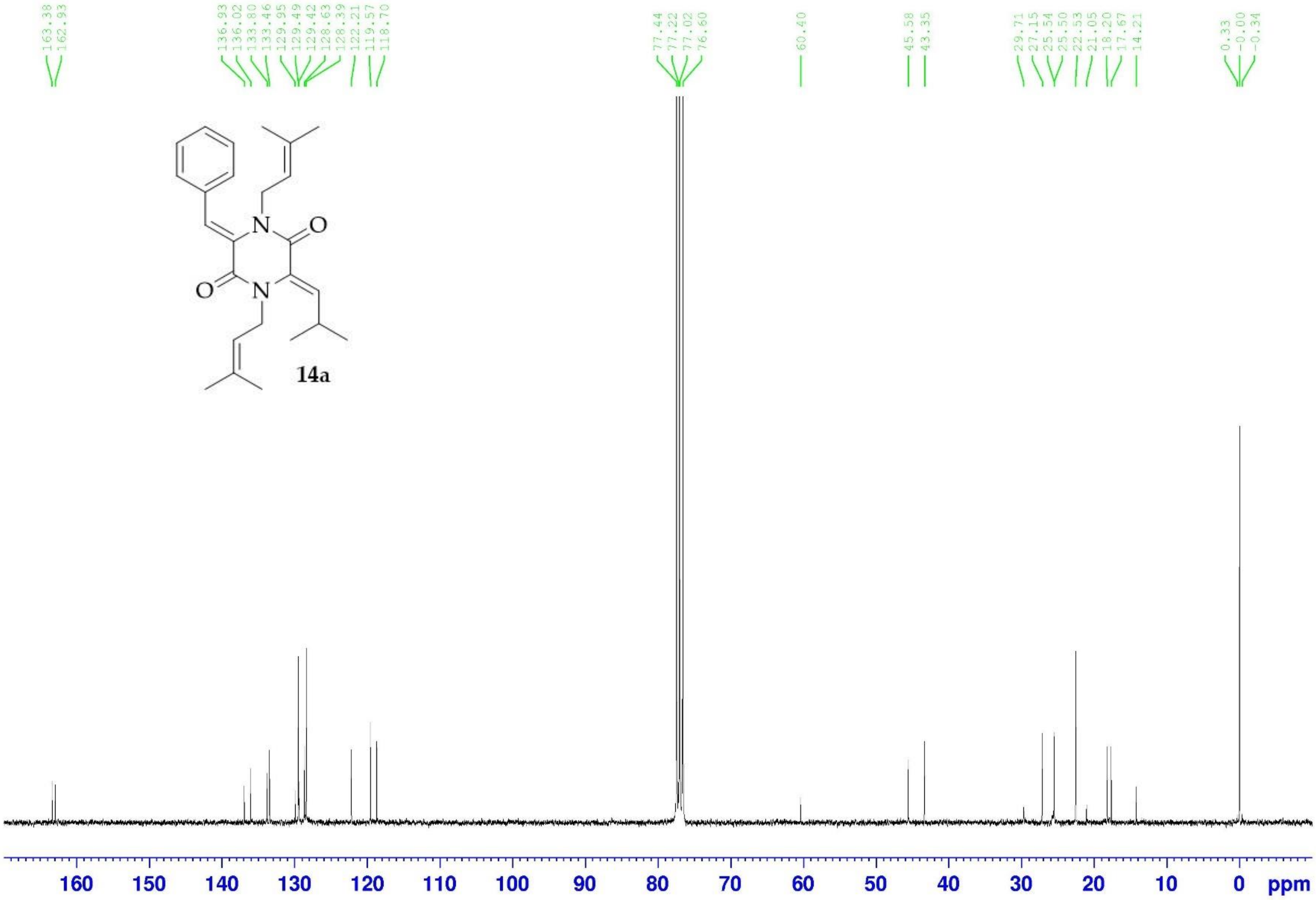

Figure S32. ^{13}C-NMR of the (3*Z*,6*Z*)-3-Benzylidene-6-(2-methylpropylidene)-1,4-bis(-3-methylbut-2-en-1yl)-2,5-diketopiperazine (**14a**).

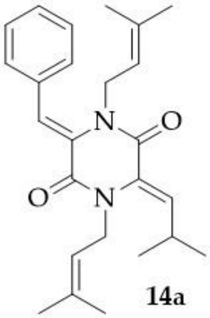

14a

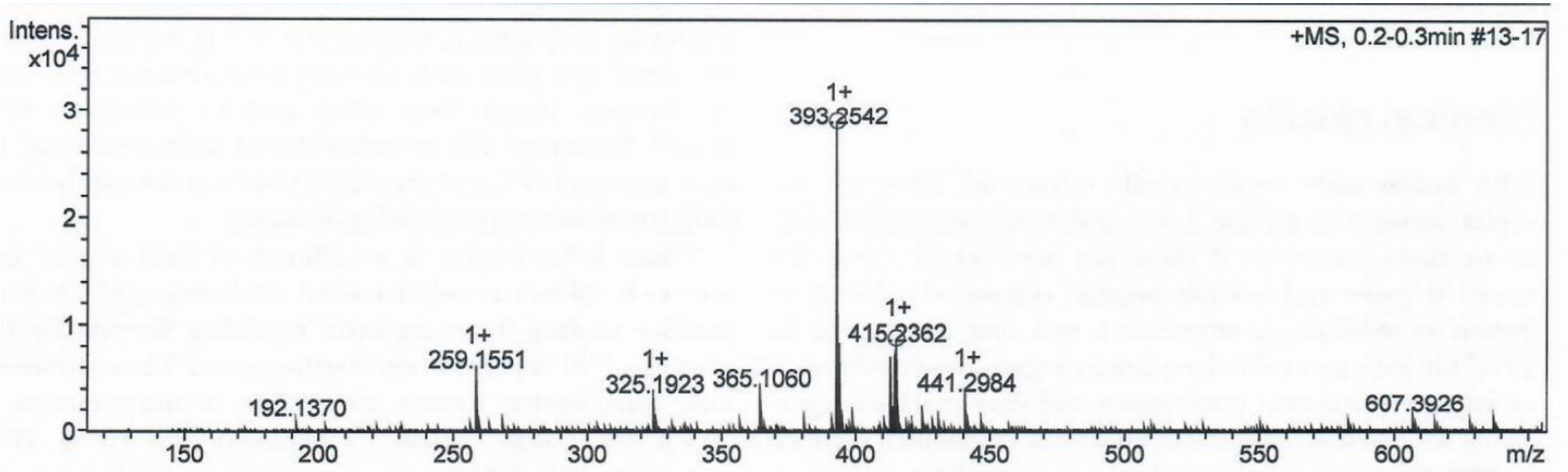

Figure S33. HRMS of the (*3Z,6Z*)-3-Benzylidene-6-(2-methylpropylidene)-1,4-bis(-3-methylbut-2-en-1yl)-2,5-diketopiperazine (**14a**).

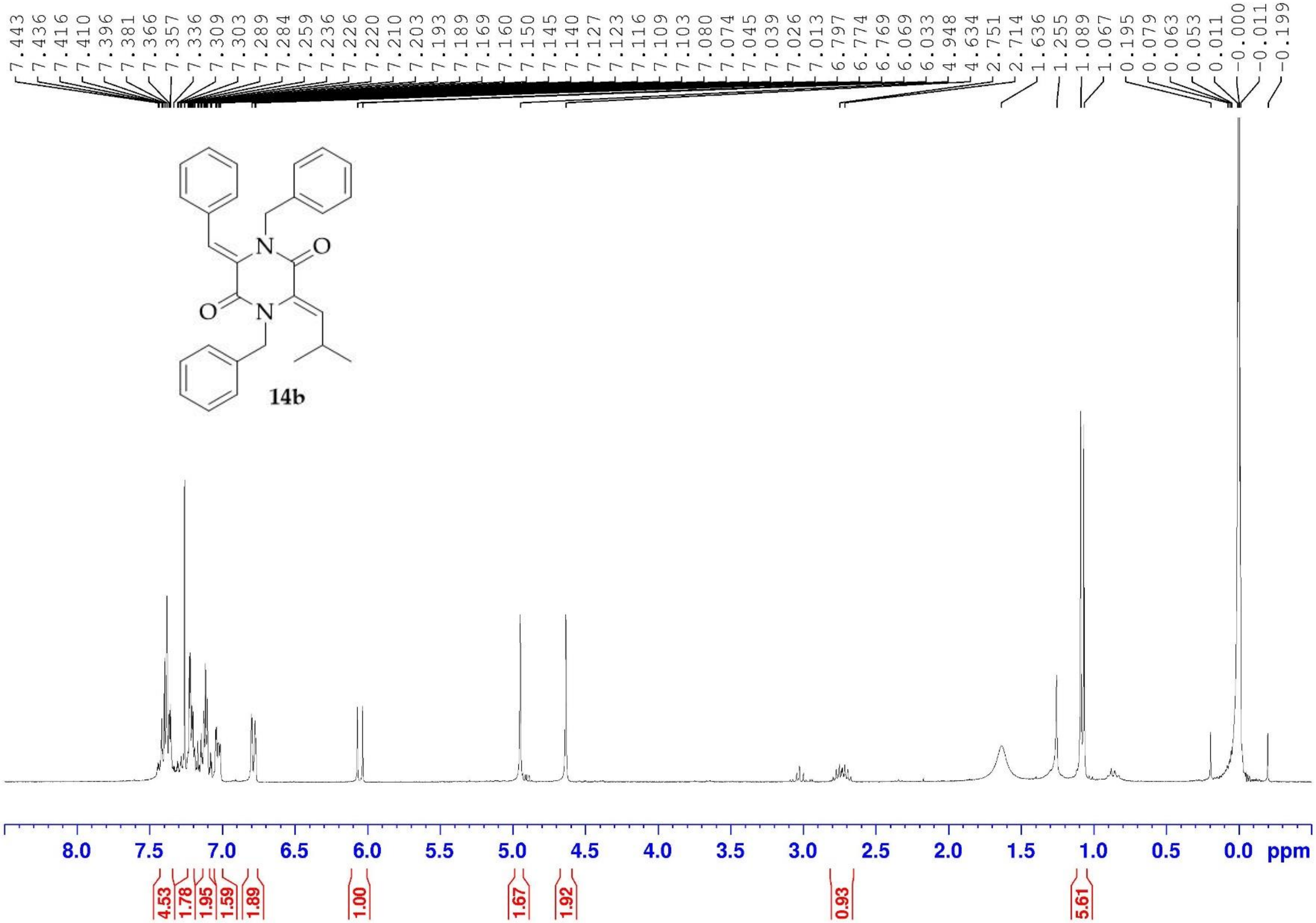

Figure S34. ^{1}H-NMR of the (*3Z,6Z*)-1,4-Dibenzyl-3-benzylidene-6-(2-methylpropylidene)-2,5-diketo piperazine (**14b**).

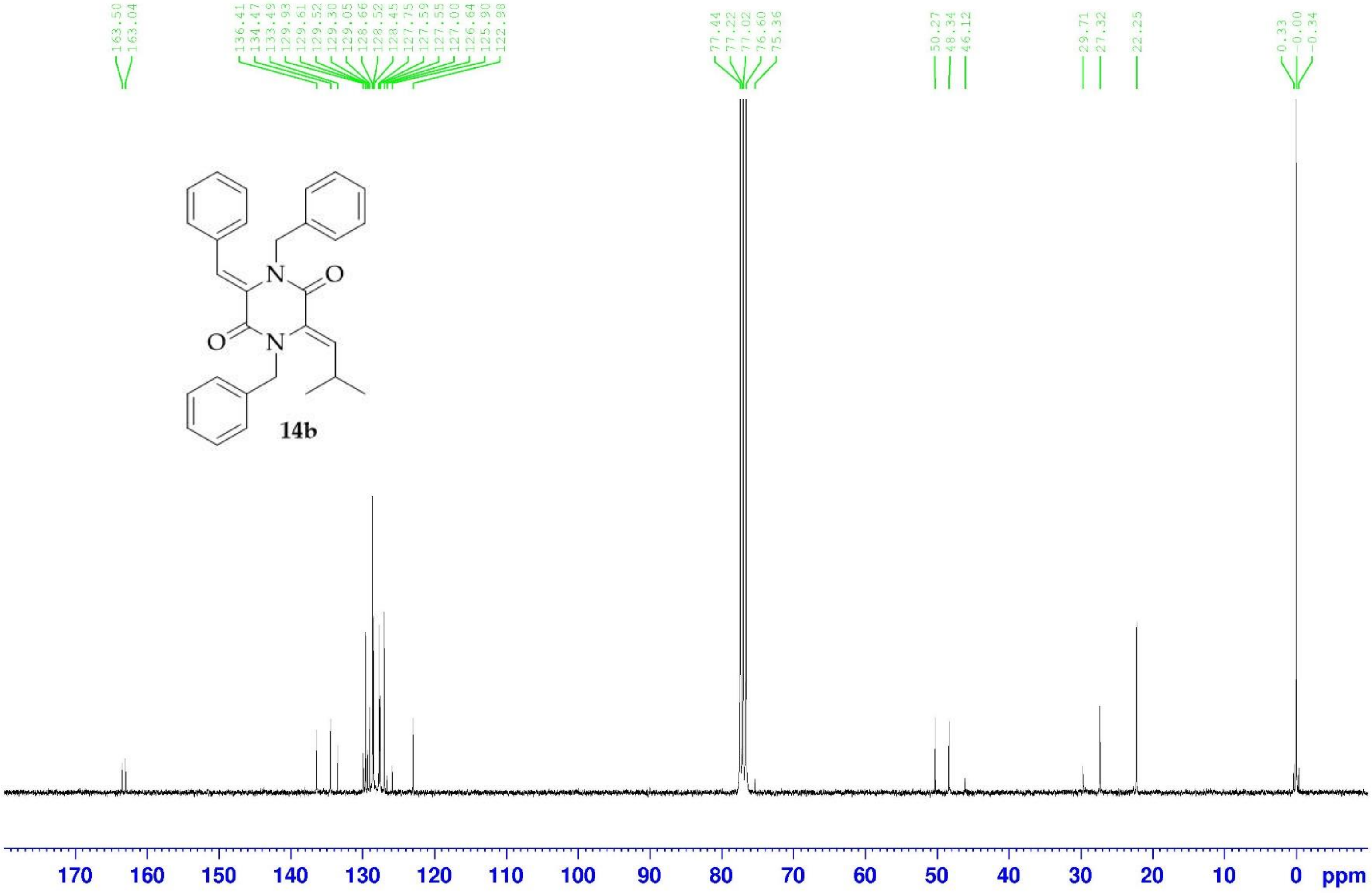

Figure S35. ^{13}C-NMR of the (*3Z,6Z*)-1,4-Dibenzyl-3-benzylidene-6-(2-methylpropylidene)-2,5-diketo piperazine (**14b**).

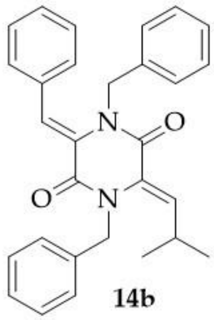

14b

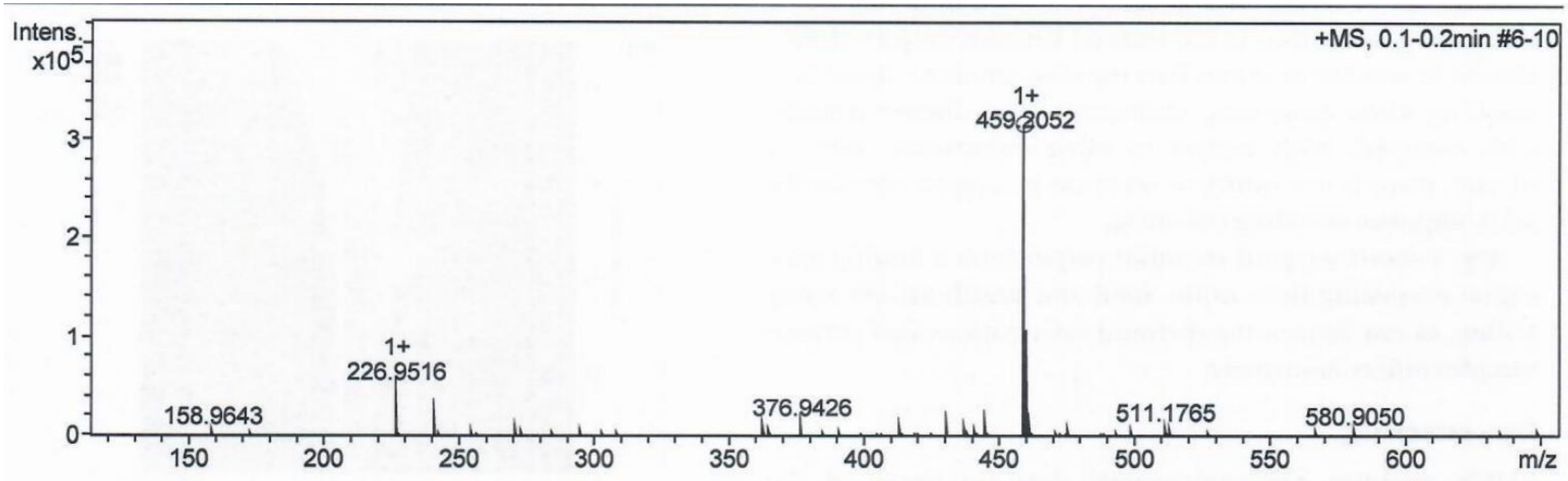

Figure S36. HRMS of the (*3Z*,*6Z*)-1,4-Dibenzyl-3-benzylidene-6-(2-methylpropylidene)-2,5-diketo piperazine (**14b**).

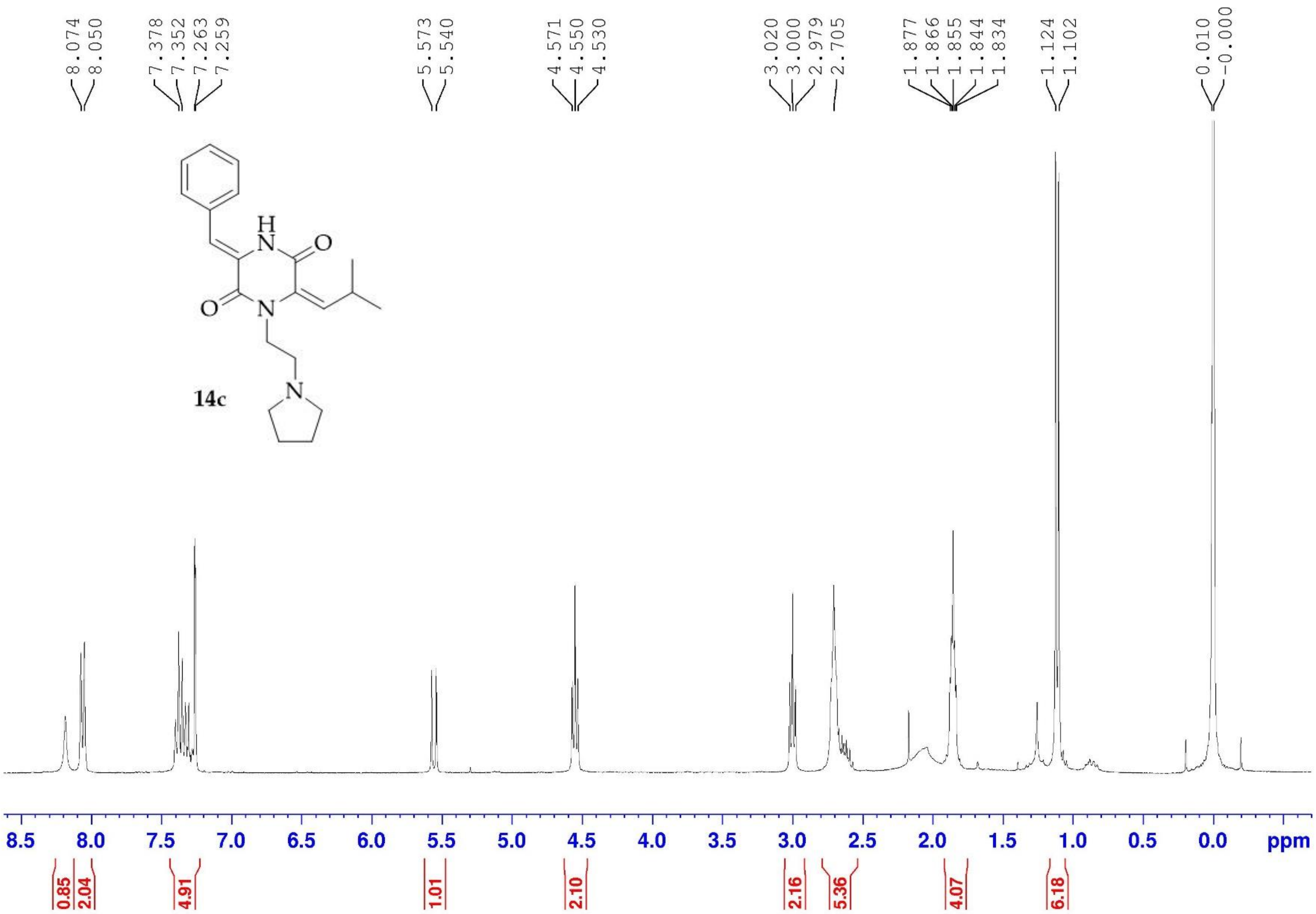

Figure S37. ^{1}H-NMR of the (*3Z,6E*)-3-Benzylidene-6-(2-methylpropylidene)-1-(1-ethyl pyrrolidine)-2,5-diketopiperazine (**14c**).

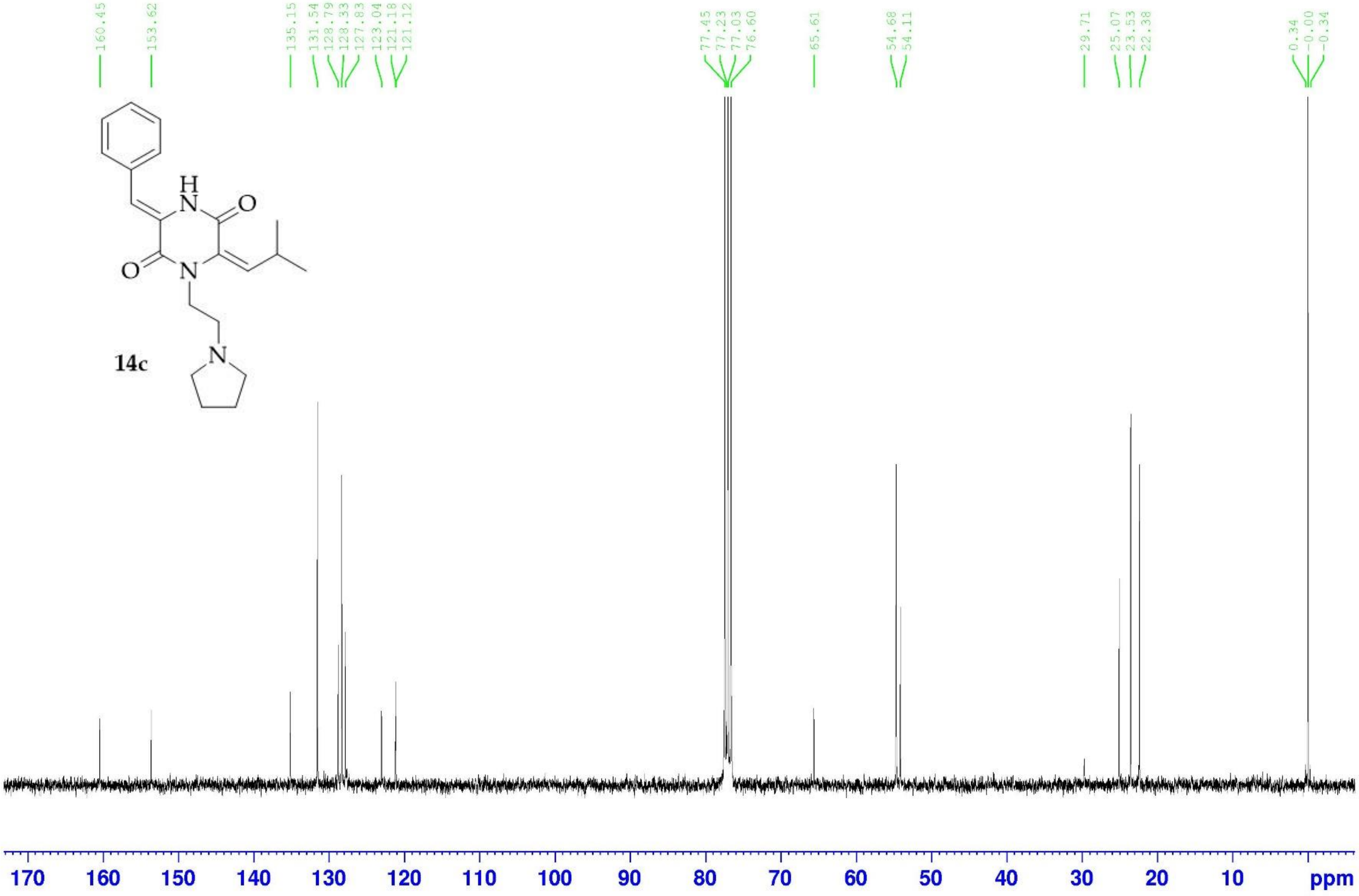

Figure S38. ^{13}C-NMR of the (*3Z,6E*)-3-Benzylidene-6-(2-methylpropylidene)-1-(1-ethyl pyrrolidine)-2,5-diketopiperazine (**14c**).

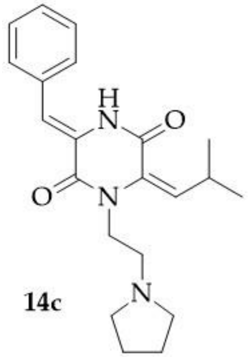

14c

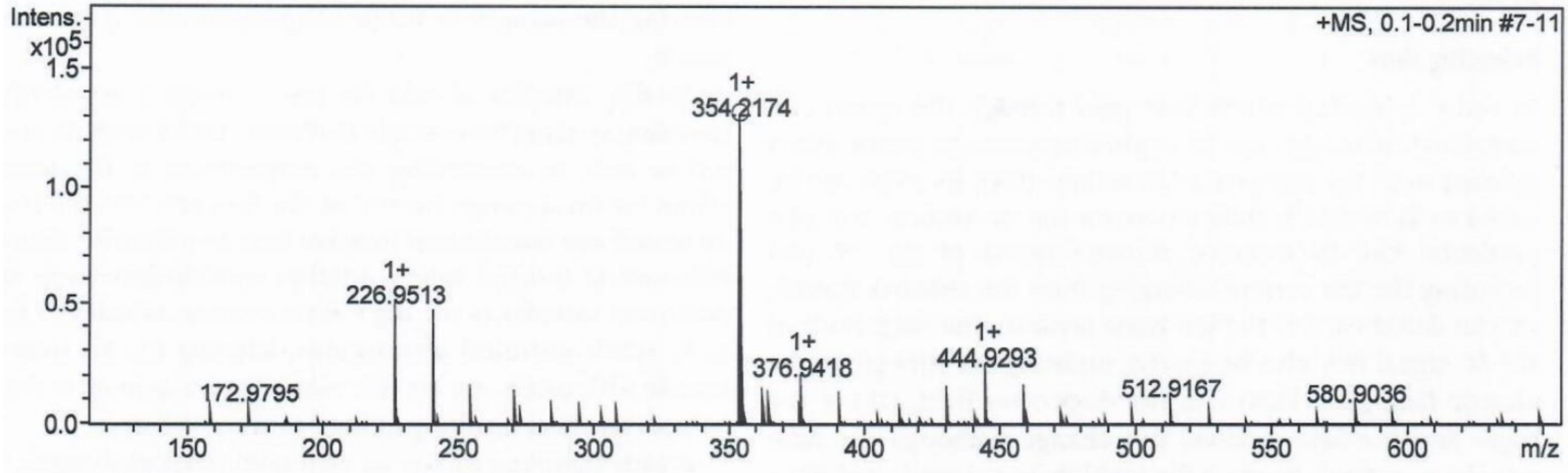

Figure S39. HRMS of the (*3Z,6E*)-3-Benzylidene-6-(2-methylpropylidene)-1-(1-ethyl pyrrolidine)-2,5-diketopiperazine (**14c**).
